# Recent Advances in Semiconductor Heterojunctions and Z-Schemes for Photocatalytic Hydrogen Generation

**DOI:** 10.1007/s41061-022-00406-5

**Published:** 2022-10-21

**Authors:** Lion Schumacher, Roland Marschall

**Affiliations:** Department of Chemistry, University of Bayreuth, 95447 Bayreuth, Germany

**Keywords:** Charge separation, Heterojunctions, Z-scheme, Photocatalysis, Hydrogen

## Abstract

The formation of semiconductor heterojunctions and Z-schemes is still a very prominent and efficient strategy of materials chemists to extend the absorption range of semiconductor combinations. Moreover, the spatial separation of photoexcited charge carriers and thereby the reduction of their recombination ultimately lead to increased photocatalytic activities. The present article reviews recent trends in semiconductor heterojunctions and Z-schemes with a focus on hydrogen generation and water splitting, exhibiting specific needs for charge carrier separation. We also included recent material trends, i.e. 2D/2D combinations, direct Z-schemes, MOFs and COFs, and combinations with upconversion materials.

## Introduction

With an estimated market of 100 million metric tons, hydrogen (H_2_) is an important basic chemical in industry and technology [1]. The worldwide most important process involving H_2_ is the production of ammonia for the production of nitrogen-based fertilisers. Steam reforming of natural gas is by far the most dominant process employed to produce H_2_ [2]. The literature describes and labels H_2_ cleanness by different colours, the three main being grey, blue, and green. The “colour” of H_2_ is determined by the source or additional technology utilised to produce the gas [3]. Since 96% of the overall H_2_ is based on fossil fuels and mainly synthesised by steam reforming/water–gas shift reactions, a vast majority of H_2_ is considered to be a polluting type of H_2_, labelled grey H_2_ [2–4]. Blue H_2_ combines grey H_2_ feedstocks with carbon capture and storage (CCS) technologies [4]. Whilst CCS technologies might defer climate change problems and buy time, H_2_ production without the emission of greenhouse gases (green H_2_) is considered to be the solution for decarbonising many large-scale chemical synthesis processes [4–6]. Promising technologies like fuel cells, the injection of H_2_ in blast furnaces and several other technologies will need further research but rely first and foremost on the large-scale production of green H_2_ [7–9]. An example of an environmentally friendly way to produce green H_2_ is photocatalytic water splitting. Challenges like low solar-to-H_2_ efficiencies as well as rare and expensive co-catalysts are yet to be solved [6]. Based on 50 years of materials research, this field has developed multiple strategies that will be essential to further improve photocatalysts in the future. Recent developments and improvements using two of these strategies—namely heterojunctions and Z-schemes—will be summarised in this review.

## State of Research

### H_2_ Production and H_2_ Economy

H_2_ can be produced from a wide range of resources using different feedstocks, pathways and technologies, including renewable resources and fossil fuels [3]. Steam reforming is by far the most dominant process employed to produce H_2_ for industrial purposes. This method uses a mix of light hydrocarbons with methane being the dominant one. In the case of natural gas, a desulphurisation step is necessary to convert sulphur into H_2_S, which is then adsorbed and removed. For the actual steam reforming, the gas mix of high-temperature steam and desulphurised feed is exposed to a nickel-based catalyst at high pressure. Depending on the process design, temperature ranges of 750–900 °C and pressure ranges of 3–25 bar are used to convert hydrocarbon and steam into synthesis gas consisting of a mix of CO and H_2_ [2]:

1 $${\text{C}}_{{x}}{{\text{H}}}_{{y}}{ + x}{\text{H}}_{2}\text{O }{ \rightleftharpoons }\text{ xCO + }\left({x + }\frac{{y}}{{2}}\right){\text{H}}_{2}$$

The steam-reforming reaction (reaction (1)) for the formation of CO and H_2_ is endothermic and therefore requires heat. External heating can be avoided or reduced by using a compact design and the exothermic partial oxidation of the feedstock (reaction (2) and (3)) [2]:

2 $${\text{C}}_{{x}}{{\text{H}}}_{{y}}{ + }\left(\frac{{x}}{{2}}{ + }\frac{{y}}{{4}}\right){\text{O}}_{2} \, { \rightleftharpoons }\text{ xCO } + \frac{{y}}{{2}} \, {\text{H}}_{2}\text{O }\text{(thermal reaction)}$$

3 $${\text{C}}_{{x}}{{\text{H}}}_{{y}}{ + }\frac{{x}}{{2}}{\text{O}}_{2} \, { \rightleftharpoons }\text{ xCO } + \frac{{y}}{{2}} \, {\text{H}}_{2} \quad (\mathrm{catalytic\, reaction})$$

However, efficient heat recovery is essential to enable an economic operation of the process. The H_2_:CO ratio in the resulting synthesis gas can be adjusted via the water–gas shift reaction (reaction (4)), which is exothermic for the formation of CO_2_ and H_2_ and allows lower temperatures than the steam reforming reaction [2].

4 $${\text{CO}}{ + }{\text{H}}_{2}\text{O }{ \rightleftharpoons } \, {\text{CO}}_{2}{ +\text{ H}}_{2}$$

Depending on the subsequent use, the gas mix is subjected to separation processes like pressure swing adsorption (PSA), membrane processes, or methanation of left-over CO.

According to reactions (1) and (4), steam reforming can produce up to four units of H_2_ gas for each unit of methane. While natural gas is the most common feedstock for steam reforming, other fossil fuels can usually be converted to lighter hydrocarbons. Gasification can be applied to all solid and liquid carbon-containing feedstocks, including biomass. As a result, the gasification of coal has a significant contribution to global greenhouse gas emissions considering the coal-based production of ammonia and methanol [2, 10].

Only a small amount of the overall produced H_2_ stems from electrochemical processes. Nonetheless, electrolysis might be a key technology since it allows the production of pure H_2_ from water while using electrical energy from renewable energy sources like wind power or photovoltaic. One distinguishes between alkaline, PEM (proton-exchange membrane), and high-temperature electrolysis. Alkaline electrolysis is a well-established production method reaching high overall efficiencies in the order of 70–80%. At temperatures between 60 and 90 °C, H_2_ is co-generated with oxygen (O_2_) [2]. Water splitting electrolytic processes are closely related to the respective fuel cell technology. High-temperature electrolysis, for example, uses the principles of inverted solid oxide fuel cells (SOFC). The energy required for the process is partially supplied as heat, leading to a lower electricity consumption compared to the other mentioned processes [2, 11].

In comparison, the price of electricity and natural gas largely defines the competitiveness between steam reforming and electrolysis. The production of H_2_ via alkaline electrolysis is often considerably more expensive than the production by steam reforming [2, 11].

While electrolysis is a well-known method to purposely produce H_2_, other chemical or electrolytic processes produce H_2_ as a side product. One example is the electrolytic production of chlorine. Common processes use membrane or diaphragm cells and produce H_2_ at the cathode. In this context, the co-production of H_2_ is often seen as a waste of energy and a safety hazard. Therefore, present developments aim at process modifications that reduce or eliminate H_2_ co-production. O_2_ depolarised cathodes are, for example, used since the beginning of this millennium. Here, O_2_ is reduced together with water to hydroxide ions, which enables a reduction in cell voltages of up to 30% [2, 12, 13].

H_2_ is used for fertiliser production, petrochemical refining, metal work, food processing, power generator cooling in power plants, and semiconductor manufacturing [3]. Hence, H_2_ is a basic substance for many industrial processes in the twenty-first century. Additionally, the term “H_2_ economy” is often used to describe ideas and challenges about a future economy heavily relying on H_2_ [4, 14–17]. The concept of H_2_ economy was originally created by John Bockris in the 1970s. It described a vision in which H_2_ is produced via water electrolysis, transported via pipelines and eventually converted back to electricity in fuel cells [18]. The idea of using H_2_ as a fuel dates back to the beginnings of fuel cell developments by W.R. Grove in 1839. Even science fiction writings from the nineteenth century discussed H_2_/water as an energy source (Jules Verne, “The Mysterious Island”) [14]. Nowadays, there are still different opinions on whether H_2_-based technologies will play a major role in a certain sector or will be outperformed by other technologies. Nevertheless, recurring statements about the future role of H_2_ and its production are found in literature. While publications from the early 2000s still partially discuss a H_2_ economy based on fossil fuels, nuclear reactors and renewable energy sources, publications from the last decade focus mainly on green H_2_ [4, 14]. Green H_2_ is seen as a solution to decarbonise many large-scale chemical synthesis processes [4]. It is also seen as an energy carrier and a storage medium for the intermittency of many renewable resources [15]. In this context, decentralisation and microgrids are discussed [4]. At the same time, H_2_ is often no longer evaluated in isolation but in conjunction with various alternatives. The term “H_2_ economy” may therefore be misleading but illustrates that (green) H_2_ will play an important role to enable a society supported entirely by renewable energy [4, 14–17].

### Photocatalytic Water Splitting

With growing attention being paid to reducing greenhouse gas emissions, renewable resources rapidly gain potential as a clean source to produce renewable H_2_ as a carbon-emissions-free energy carrier [3]. Solar energy can be seen as the most abundant renewable energy source. Therefore, H_2_ production from solar energy is considered to be a promising solution for sustainable energy [19].

**Fig. 1:**
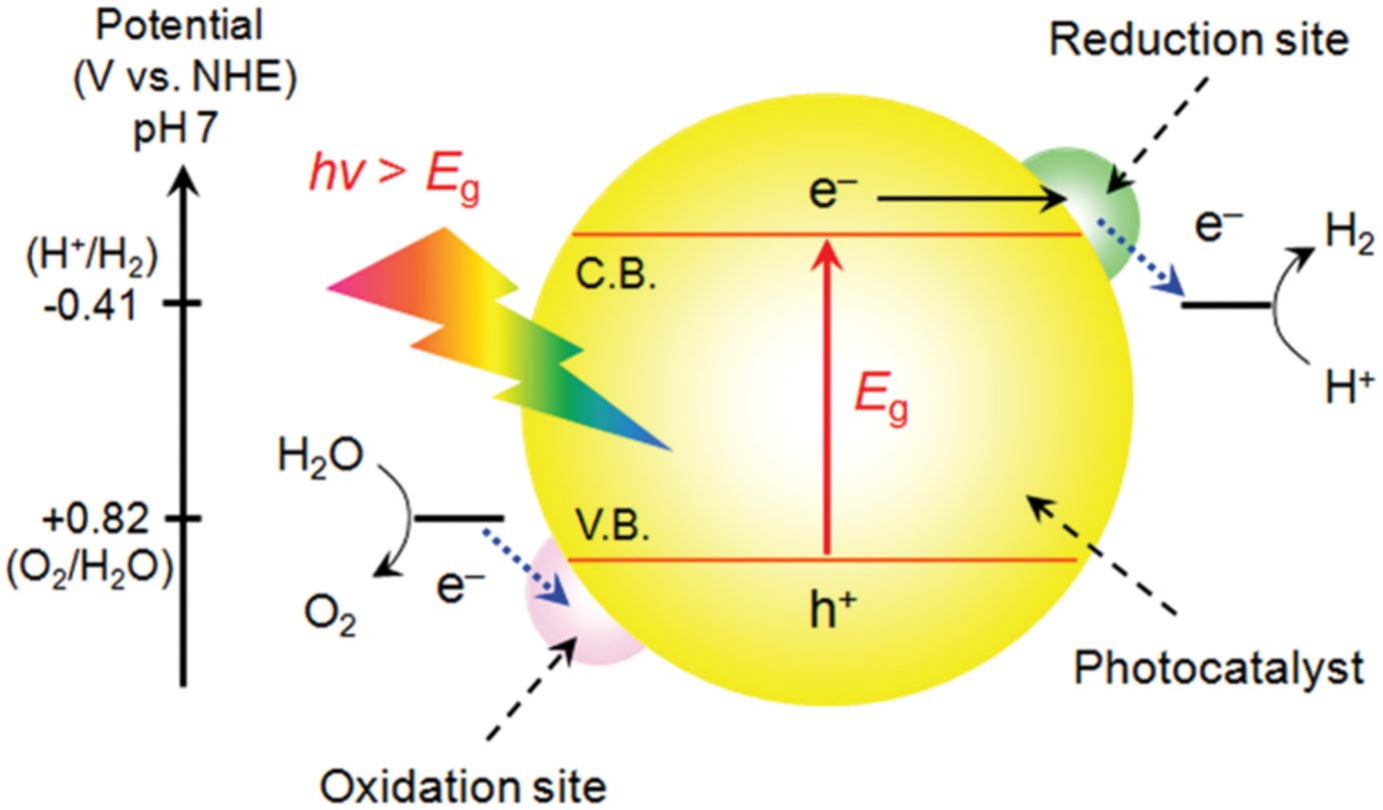
Schematic energy diagram of photocatalytic water splitting; oxidation site and reduction site might refer to co-catalysts discussed in the next section. Reprinted with permission from [24] © 2010 American Chemical Society

Semiconductor materials can convert sunlight energy into chemical energy by catalysing the formation of chemical bonds. Thus, generating H_2_ and O_2_ by performing photocatalytic water splitting is intensely investigated [20–23]. A semiconductor absorbs sunlight when the energy of the incident photon is equal to or larger than the band gap (*E*_g_). An electron is thereby excited from the valence band (VB) into the conduction band (CB) of the semiconductor (Fig. 1). Together with the remaining hole in the VB, an exciton is formed [20].

The photoexcited electron can be used to reduce protons to H_2_ if the CB minimum has a more negative potential than the electrochemical potential of reaction (5). Similarly, the photogenerated hole in the VB can perform the electrochemical oxidation of water to O_2_ if the maximum of the VB is more positive than the electrochemical potential of reaction (6) [20, 23]:

5 $${2}{\text{H}}^{+}{ \,+ \,}{2}{\text{e}}^{-}{ \to }{\text{H}}_{{2(g)}}\quad \Delta {\text{E}}^{0} \, = +0.41\,\mathrm{ V \,at\, pH }\,7$$

6 $${\text{H}}_{2}{\text{O}}{ \,+\, }{2}{{h}}^{+}{ \to 0.5}{\text{O}}_{{2(g)}}+ { 2} {\text{H}}^{+}\quad \Delta {\text{E}}^{0} \, = +0.82\,\mathrm{ V\, at\, pH }\,7$$

Compared to the H_2_ evolution reaction (HER), the O_2_ evolution reaction (OER) is comparatively more complex because this reaction requires a four-electron oxidation step combined with the removal of four protons to form a relatively weak oxygen–oxygen bond [25]. The overall water splitting reaction is an uphill reaction with a positive shift in Gibbs free energy (+ 237 kJ/mol). Therefore, water splitting is thermodynamically a photosynthetic reaction [26]. In theory, the minimum band gap energy (*E*_g_) of the semiconductor should be 1.23 eV (approximately equivalent to a wavelength of 1000 nm). Due to energy losses, kinetic overpotentials are needed; thus, the *E*_g_ of a single semiconductor should lie in the range of 1.5–2.5 eV [23].

It is known that the CB potential of a semiconductor material in aqueous solutions usually exhibits a pH dependence according to the following equation [23]:

$${\text{E}}_{{{\text{CB}}}} = {\text{E}}^{0}_{{{\text{CB}}}} ({\text{pH }}0)-0.0{\text{59 pH}}$$

Since the redox potentials of water show the same linear dependence with a slope of 0.059 V per pH, band edge positions of the semiconductor usually cannot be shifted relative to the redox potentials of water by changing the pH value [23].

### Strategies for the Improvement of Photocatalysts

#### Single Absorber, Co-catalysts and Sacrificial Agents

So far, many semiconductors have been found to be capable of producing H_2_ or O_2_ under light irradiation [23, 27, 28]. Thereby, metal oxide semiconductors can usually be divided into two classes: materials containing metal cations with *d*^*0*^ configuration and those with *d*^10^ configuration [6, 23]. Many recent research activities are focused on composite materials, but single absorbers are still investigated. This is due to newly discovered materials or known materials that are back in the focus of research. One example is SrTiO_3_ which experienced a renaissance due to new insights into defect engineering and the electronic structure of doped SrTiO_3_ [6, 29–31].

Charge carrier recombination limits photocatalytic efficiencies in many photocatalytic systems but especially for undecorated semiconductors used as photocatalysts. Increasing the crystallinity of a photocatalyst can reduce recombination. Defects and grain boundaries are related to interband and surface states, which act as traps for holes. Therefore, increasing the crystallinity can result in higher photocatalytic efficiencies [20]. Another possibility to prevent charge carrier recombination is charge separation. One popular strategy is the use of co-catalysts often applied as nanoparticles on the surface of a semiconductor photocatalyst. The Fermi energy of the metal nanoparticle is usually lower than that of the semiconductor, facilitating electron transfer from the semiconductor to the metal via a Schottky contact [20]. Usually noble metal nanoparticles such as rhodium, palladium, platinum or gold are used. Noble metals serve as electron sinks, thus spatially separating the electron from the photoexcited hole in VB of the semiconductor [20, 32]. Besides noble metals as co-catalysts for the reduction reaction, other materials like metal oxides (RuO_2_, NiO, CuO/Cr_2_O_3_, Rh_2-y_Cr_y_O_3_), metal sulphides (MoS_2_) and molecular co-catalysts have been investigated [20, 32–35]. For the oxidation reaction, metal oxides such as RuO_2_ and IrO_2_ are used. Efforts are being made to use more earth-abundant alternatives for both the reduction and the oxidation reaction [20, 32]. Charge carrier separation due to co-catalysts can also stabilise certain photocatalysts since some sulphides, oxysulphides, oxynitrides and nitride semiconductors are known for their photoinstability, which is usually due to photocorrosion. Therefore, loading of oxidation cocatalysts like PdS can protect semiconductors that tend to be oxidised by photogenerated holes [32, 36].

**Fig. 2:**
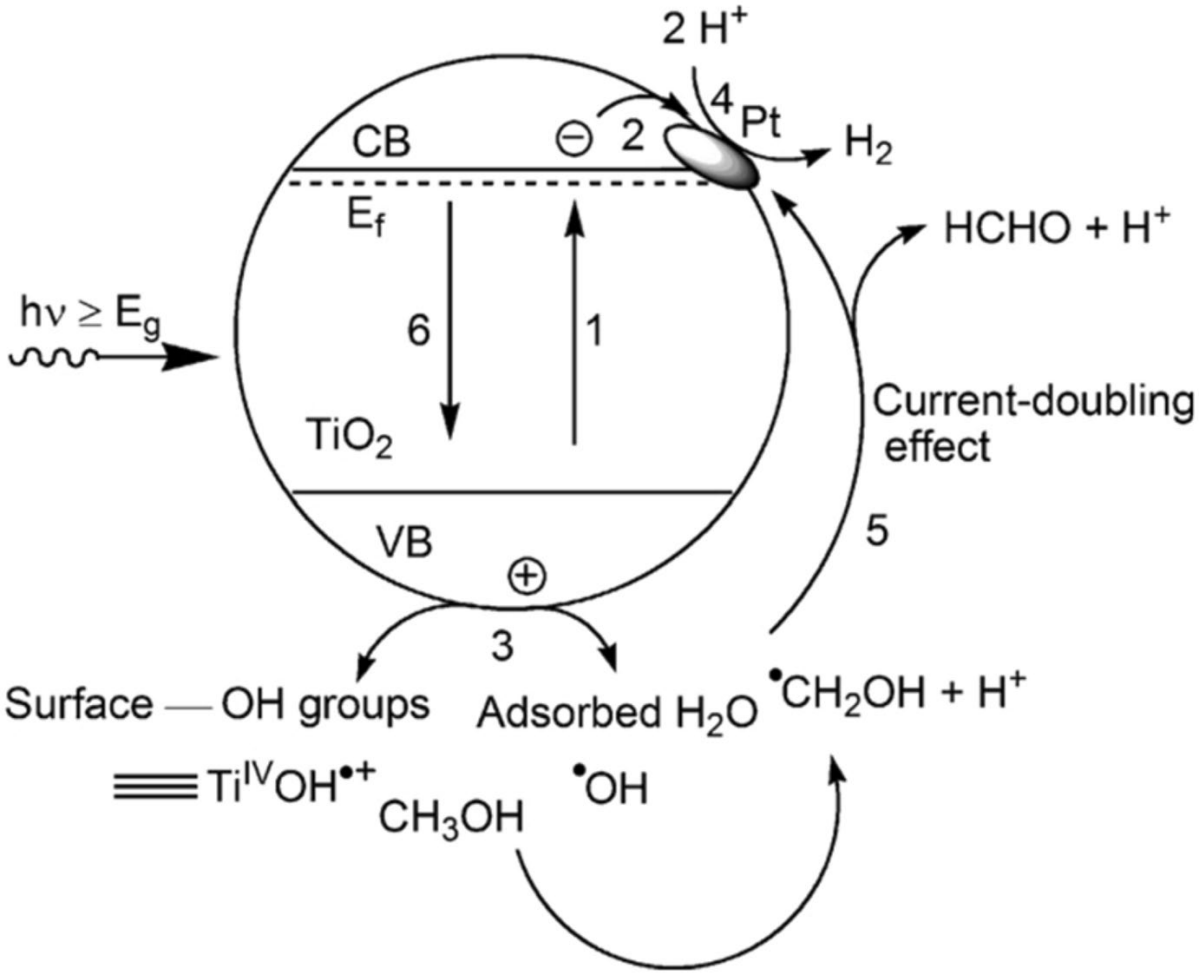
Schematic representing the proposed steps for the photocatalytic molecular H_2_ production from aqueous methanol solution, (1) photogeneration of charge carriers, e^−^ and h^+^; (2) trapping of e^−^ by Pt islands; (3) first oxidation step of CH_3_OH by either trapped hole or hydroxyl radical, ^**·**^OH; (4) reduction of H^+^; (5) formation of HCHO through e^−^ injection into the conduction band of TiO_2_ or to the Pt islands (current-doubling); (6) recombination channel. Reprinted with permission from [39] © 2011 Elsevier

As already mentioned, the reduction and oxidation of water is a complex multistep reaction involving four electrons. Thus, photocatalytic water splitting is a rather inefficient process and far away from large-scale industrial applications. Using electron donors can improve the H_2_ production as holes are scavenged by these molecules. Additionally, charge carrier recombination can be reduced and the back reaction to water is supressed because O_2_ is not produced [37]. Various organic and inorganic compounds (alcohols, organic acids, hydrocarbons, sulphides and sulphites) are being employed as hole scavengers/electron donors [28, 37]. Methanol is one of the most frequently used sacrificial agents. Nonetheless, the application of methanol is highly debatable for environmental reasons even if the substance is derived from biomass. Furthermore, molecular H_2_ formation in systems using sacrificial agents should not be called water splitting. The term water splitting should only be used when pure water is used as solvent and reactant, without any additional reagents [6]. Acting as an electron donor, methanol reacts irreversibly with the photogenerated VB holes (Fig. 2) [37]. In the overall methanol decomposition reaction (not shown in Fig. 2), methanol can be stepwise oxidised to carbon dioxide [38].

Similarly, sacrificial electron acceptors can be used to support the oxidation of water. Silver cations, Ag^+^, are employed by a vast majority of research groups. The photocatalytic formation of molecular O_2_ is accompanied by the reduction of Ag^+^ and thereby accompanied by the deposition of metallic silver nanocontacts. The optical changes and changes in catalytic activity arising from this deposition should always be discussed [37].

#### Heterojunctions

Key issues for improving photocatalytic activity are improved light absorption, high crystallinity, large surface areas, and effective charge separation. Furthermore, suitable band positions are needed, as well as earth-abundant elements and high chemical stability of the photocatalyst [20]. A composite photocatalyst system consisting of two or more semiconductors allows different favourable properties from each participating compound to be combined, extending the absorption range of the visible spectrum, reducing photoexcited electron hole recombination and increasing the photo-corrosion stability, thus improving water splitting efficiency [40–43].

**Fig. 3:**
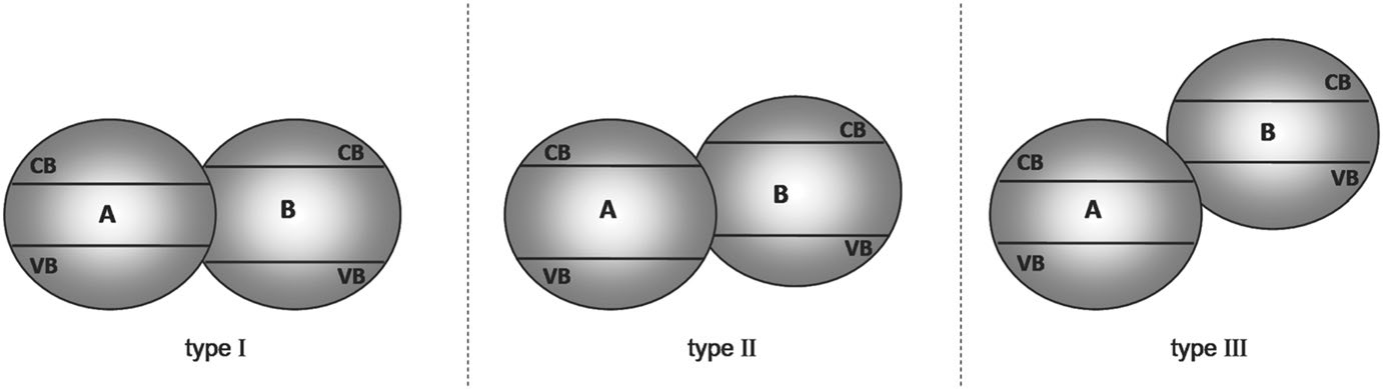
Different types of semiconductor heterojunctions. Reprinted with permission from [20] © 2013 Wiley-VCH

Heterojunctions formed by two semiconductors can be classified into three different types depending on the band position (Fig. 3).

In type I heterojunctions, material A has a smaller band gap than material B. Due to a more negative CB and a more positive VB of material B, holes and electrons transfer from material B to material A. An example of a type I heterojunction is TiO_2_/Fe_2_O_3_ where photogenerated electron and hole flow occurs from TiO_2_ to Fe_2_O_3_ [40, 44, 45]. A type I heterojunction yields therefore no improvement to charge carrier separatio and thus, in theory, no improvement in photocatalytic activity. Due to many wide band gap semiconductors known and limitations in valence band and conduction band positions, type I heterojunctions are common when composite materials consist of a small band gap and a wide band gap semiconductor [20, 40, 46].

In type II heterojunctions, the CB position of material B is more negative than that of material A, while material A has a more positive VB position. As a result, electrons and holes transfer in opposite directions, which leads to improved charge carrier separation, reduced recombination probability, increased charge carrier lifetimes, and, in the end, to improved photocatalytic activity. Most of the examples of composite photocatalysts described in the literature are type II heterojunctions. A WO_3_/BiVO_4_ heterojunction is one example of this type of system [20, 40, 47].

In type III heterojunctions the charge carrier transfer is the same as in type II semiconductors, but the band positions are further set off. Due to the band position, these systems are also called broken-gap situations [20]. A BP/ReS_2_ system (BP = black phosphorus) is one example of this type. In general, type III heterojunctions are rather rarely reported but for example discussed for electronic applications [48].

##### Recent Trends in Heterojunction Photocatalysts

Tremendous efforts have been dedicated to the development of type II heterojunction photocatalysts due to their ability to separate photogenerated electrons and holes. However, in the past few years, a trend towards Z-schemes (next section) can be observed. Broad review articles discussing improvements in heterojunction photocatalysts have usually been written in 2017 or earlier [20, 47, 49, 50]. Recent review articles are rare and heterojunctions are rather discussed in articles that only cover one specific material. However, there are recent trends that are worth mentioning.

Photocatalysts made of 2D materials are of particular interest due to their electrical and optical properties. Type II heterojunctions based on 2D materials have been designed and studied as efficient photocatalysts theoretically and experimentally [51–55]. Hua et al*.*, for example, reported a 2D La_2_Ti_2_O_7_/In_2_S_3_ type II heterojunction [52]. The intimate contact of the two layers was realised by strong Coulomb static forces due to inverse zeta potentials of both components. The strong interaction between the two layers and the type II heterojunction co-promoted the charge separation, resulting in H_2_ production rates 3.5 times higher than pristine In_2_S_3_ and 18 times higher than the physical mixture of both components [51, 52]. Although type II heterojunctions can separate photoinduced charges efficiently, *p-n* heterojunctions can separate the charges even faster with the aid of internal electric fields [51].

In 2018, Qin et al. constructed a *p-n* heterojunction by attaching *p-*type Cu_3_P nanoparticles to the surface of *n-*type g-C_3_N_4_. The photocatalyst exhibited 95 times higher hydrogen evolution activity from 10 vol% triethanolamine (TEOA) solution than bare g-C_3_N_4_ with an apparent quantum efficiency of 2.6% at 420 nm [56]. Two years later, a MoS_2_/Bi_2_O_3_ *p-n* heterojunction for overall water splitting was described. Bi_2_O_3_ nanorods were anchored on MoS_2_ microflowers. As a result, H_2_ evolution rates increased by a factor of ten [57].

Qin et al. prepared a 2D/2D g-C_3_N_4_/ZnIn_2_S_4_ heterojunction photocatalyst [58]. The improved photocatalytic performance (6.095 mmol g^−1^ h^−1^ using triethanolamine as sacrificial agent) was attributed to the larger contact area and sulphur vacancies that acted as active sites for trapping electrons, which led to elongated charge lifetimes. Furthermore, the vacancies shortened the band gap, enhancing the absorption of visible light. Van der Waals (vdW) forces acted as an intermolecular driving force for charge carrier transport. This was considered to be an alternative route for enhancing H_2_ evolution efficiency since heterojunctions are therefore not restricted by the lattice matching of the component materials [58].

**Fig. 4:**
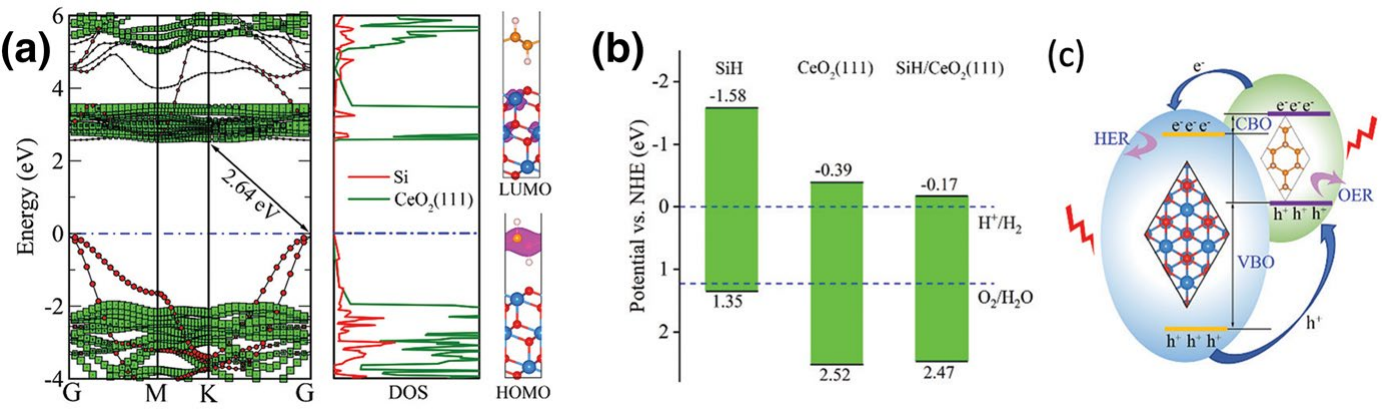
**a** Electronic structure of the SiH/CeO_2_(111) heterojunction. The projected band structure (left panel), partial density of states (PDOS, middle panel), as well as the highest occupied molecular orbital (HOMO) and lowest unoccupied molecular orbital (LUMO) (right panel). The green squares and red circles in the left panel represent the CeO_2_(111) and SiH constituents, respectively. The Fermi level is set to zero. **b** Calculated VBM and CBM potentials versus normal hydrogen electrode (NHE) of SiH, CeO_2_(111), and SiH/CeO_2_(111) heterojunction. The upper and lower blue dashed lines stand for the proton reduction potential (H^+^/H_2_) and O_2_ reduction potential (O_2_/H_2_O) for water splitting with values of 0 and 1.23 eV (pH 0), respectively. **c** Schematic diagram of charge transfer between SiH/CeO_2_(111) heterojunction layers. Reprinted with permission from [59] © 2021 Royal Society of Chemistry

A similar approach was used by Zeng et al. for theoretical description of a SiH/CeO_2_(111) type II heterojunction [59]. Both components were chosen because of a < 1% lattice mismatch. The staggered band structure (Fig. 4) demonstrates that SiH/CeO_2_(111) is a type II vdW heterojunction, which effectively promotes the separation of photogenerated hole-electron pairs and should improve its photocatalytic activity.

The band gap of the SiH/CeO_2_(111) heterojunction is 2.64 eV and thus smaller than the band gap of the SiH monolayer and CeO_2_(111). Light absorption of the composite material should be significantly extended into the visible light wavelength range compared to that of pure CeO_2_(111), and oxygen vacancies on the surface of CeO_2_(111) can also enhance its visible light absorption performance. Moreover, valence band maximum (VBM) and conduction band minimum (CBM) potentials meet the requirements of water splitting (Fig. 4) [59]. Simulations like these might help to synthesise deliberately chosen composite materials in the future.

**Fig. 5:**
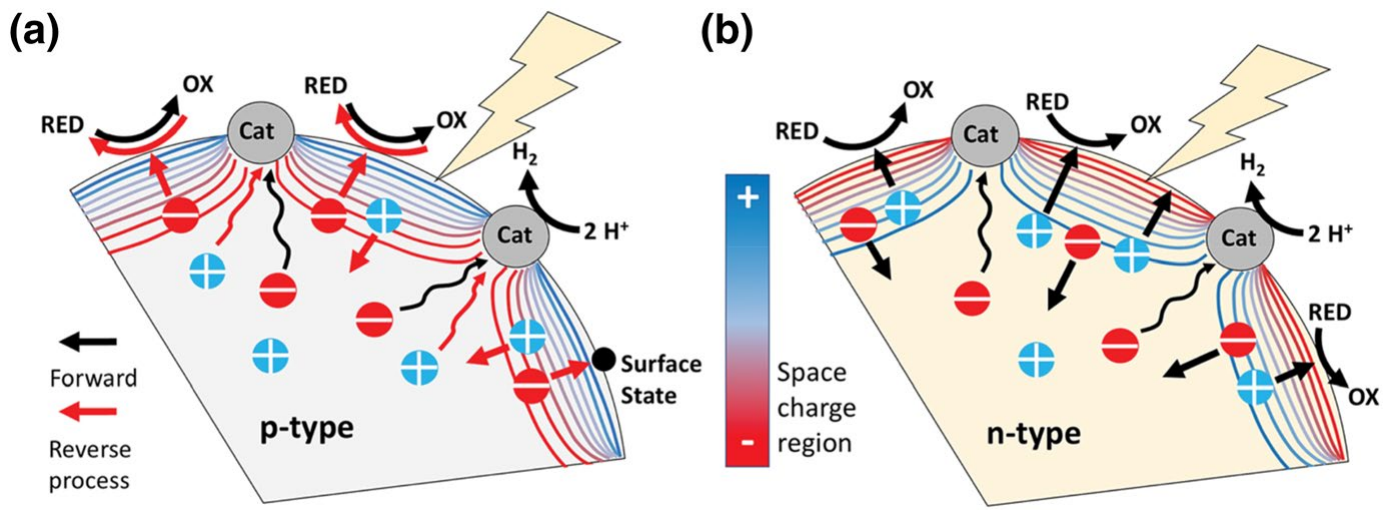
Depletion layer and charge carrier movement in illuminated **a** *p*-type and **b** *n*-type semiconductor/co-catalyst configurations in aqueous solutions of a reducing sacrificial agent. Straight arrows indicate drift and wavy arrows indicate diffusion. Minority carriers (holes for *n*-type SC and electrons for *p*-type SC) generated inside the depletion layer are attracted to the surface and away from the proton reduction co-catalyst (Cat). For *p*-type semiconductors this promotes the back reaction (red arrows) and for *n*-type semiconductors the forward reaction (black arrows). Reprinted with permission from [60] © 2021 Royal Society of Chemistry

Deep investigations might explain and solve problems that have limited the performance of (heterojunction) photocatalysts in the past. For example, it is known that certain *p*-type materials can be used as photocathode materials for H_2_ evolution, but only show low activity as photocatalysts. Zhao et al. investigated *p*-type gallium phosphide to better understand this limitation [60]. H_2_ evolution rates could be inversely correlated with the standard reduction potential of the used donors (KI, K_4_[Fe(CN)_6_], Na_2_SO_3_; more reducing donors give lower rates). Due to a depletion layer at the *p*-type gallium phosphide/electrolyte interface, photogenerated electrons are directed away from the cocatalyst (here Ni_2_P) toward the sacrificial donors and surface states (Fig. 5).

The electrons react with the surface states or with the oxidised form of the sacrificial electron donor (e.g. triiodide is reduced to iodide). As a result, electrons deep inside the *p*-type semiconductor must reach the cocatalyst by diffusion. This is a slow process because electrons are the minority carriers with a short lifetime. According to surface photovoltage measurements, the depletion layer had a barrier height of up to 0.45 eV for Na_2_SO_3_. Findings like these explain basic observations in semiconductor photocatalysis and might play a key role in the development of *p*-type and composite photocatalysts [60].

In recent years, the number of examples for heterojunctions with three type of absorber materials has significantly increased. One of the earliest examples for such a triple heterojunction was the combination of anatase-TiO_2_/rutile-TiO_2_/WO_3_ [61]. Instead of WO_3_, Huang et al. used carbon nitride in combination with anatase-TiO_2_ and rutile-TiO_2_, increasing the visible light absorption further for H_2_ and O_2_ evolution [62]. Other triple absorber heterojunctions reported including TiO_2_ are for example Fe_2_TiO_5_/Fe_2_O_3_/TiO_2_ [63], Ag_2_O/Fe_2_O_3_/TiO_2_ [64], and Bi_2_O_3_/C_3_N_4_/TiO_2_ [65].

An interesting example is the combination of CoP/CdS/WS_2_, containing no oxide at all forming a *p-n-n* heterojunction for H_2_ generation [66]. The apparent quantum efficiency at *λ* = 420 nm was reported to be 1.34%; unfortunately, no oxidation product was confirmed in pure water. Containing two other sulphide materials, ZnS/CdS/TaON heterojunctions were reported to show 14 times higher H_2_ evolution activity (from sulphide/sulphite solutions) compared to CdS/TaON, in the absence of noble-metal co-catalysts and an optimum amount of ZnS of 6 wt% [67]. Overall, 839.6 μmol h^−1^ g^−1^ could be reached under AM 1.5G illumination; no quantum efficiencies were reported. Since ZnS has quite a large band gap compared to CdS and TaON, ZnS might act as a hole sink/co-catalyst rather than an absorber in such a system. CdS on the other hand is very prominent for its ideal band gap for water splitting but well-known photocorrosion issues. In combination with Bi_20_TiO_32_ and Bi_4_Ti_3_O_12,_ it was reported as ternary heterojunction for H_2_ evolution recently [68]. Photocatalytic H_2_ production (from aqueous methanol solution) of up to H_2_ production 1890 µmol g^−1^ h^−1^ under 250-W xenon lamp (*λ* > 400 nm) illumination was reported, with strongly increased activities compared to Bi_4_Ti_3_O_12_, CdS, or Bi_20_TiO_32_/Bi_4_Ti_3_O_12_. However, not a heterojunction behaviour but a scheme involving a type I bridged coupled Z-scheme system was proposed to explain the observed activities. Z-schemes will be covered in the following section.

#### Z-Schemes

**Fig. 6:**
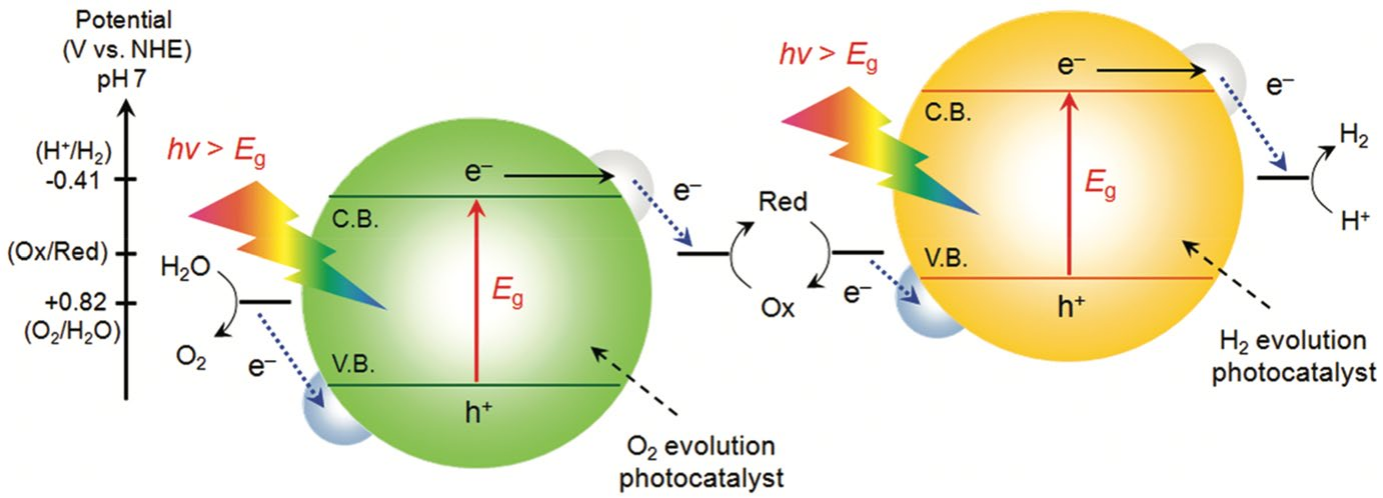
Schematic energy diagram of photocatalytic water splitting for a two-step photoexcitation system. Reprinted with permission from [24] © 2010 American Chemical Society

Z-schemes use two different semiconductors and typically a reversible donor/acceptor pair, a so-called shuttle redox mediator (Fig. 6). This system is inspired by natural photosynthesis in green plants, where photosystems I and II harvest 700 and 680-nm photons, respectively [69].

A traditional Z-scheme photocatalyst was first proposed by Bard in 1979 [70]. Since then, redox couple mediated Z-schemes have attracted considerable attention. In contrast to heterojunctions, the semiconductors are usually not in direct contact to each other but electronically coupled via the redox mediator (the next section will concentrate on solid Z-schemes). Thus, semiconductors can be combined that can only perform either water reduction or oxidation due to their band potential [69]. For example, WO_3_, which does not have the ability to reduce H^+^, is capable of producing O_2_ from an aqueous solution containing appropriate electron acceptors under visible light and acts as an effective building block for O_2_ evolution in Z-scheme water splitting [69].

The redox mediator is an essential component in the Z-scheme water splitting system because it transfers electrons from an O_2_ evolution photocatalyst to a H_2_ evolution catalyst. Therefore, the flow of charge carriers is reversed in Z-schemes compared to the above mentioned heterojunctions of two semiconductors [6]. The process leads to spatially separated, high-redox-capacity electrons and holes in the H_2_ evolving photocatalyst and the O_2_ evolving photocatalyst, respectively.

The most employed redox couples are Fe^3+^/Fe^2+^ and IO_3_^–^/I^–^, which both have unique characters affecting the efficiency of Z-scheme water splitting. The following reactions describe a water splitting system using the IO_3_^–^/I^–^ couple as a mediator [69]:

7 $${{I}}^{-}{ + 3}{\text{H}}_{2}\text{O + 6}{\text{h}}^{+}{ \to }{\text{IO}}_{3}^{-}{ + }{\text{6H}}^{+}$$

8 $${2}{\text{H}}^{+}{ \,+\, }{\text{2e}}^{-}{ \to }{\text{H}}_{\text{2(g)}}$$

9 $${\text{IO}}_{3}^{-}{ + }{\text{6e}}^{-}{ + 3}{\text{H}}_{2}\text{O }{ \to }{\text{I}}^{-}{ + 6}{\text{OH}}^{-}$$

10 $${2}{\text{H}}_{2}\text{O + 4}{\text{h}}^{+}{ \to }{\text{O}}_{\text{2(g)}}+ { } {\text{4H}}^{+}$$

Taking the IO_3_^–^/I^–^ couple, there is a dependence of activity on the concentration of the redox mediator. With increasing the concentration of I^–^, the efficiency of the I^–^ oxidation by VB holes in a H_2_ evolution photocatalyst is enhanced, while the oxidation reaction in the O_2_ evolution site is suppressed because of competitive oxidation of I^–^ by photogenerated holes in the VB of the O_2_ evolution photocatalyst. Hence, using NaI as an initiator, there is a volcano-type trend between the concentration of NaI and Z-scheme activity in most cases [69, 71–73].

An Fe^2+^/Fe^3+^ redox system is limited to acidic conditions because iron ions undergo precipitation (iron hydroxide) in neutral and basic conditions. The IO_3_^–^/I^–^ couple can be employed in a wider range of pH values but also shows a pH dependence [69].

Apart from their role of shuttling electrons, redox mediators show multiple side effects in Z-scheme water splitting. One example is that I^–^ ions apparently undergo adsorption onto the surface of Pt, used as a co-catalyst, forming an iodine layer which suppresses the backward reaction of water formation [69, 74]. A similar behaviour was reported for the Fe^2+^/Fe^3+^ redox mediator. Water formation and the reduction of Fe^3+^ are efficiently suppressed by adsorption of [Fe(SO_4_)(H_2_O)_5_]^+^ and/or [Fe(OH)(H_2_O)_5_]^2+^ on Pt surfaces [69, 75]. Although the backward reaction of water formation on Pt is not completely suppressed by adsorbed iron species, further studies revealed that Ru does not suffer from water formation. Using Ru as a co-catalyst, the reduction of Fe^3+^ by H_2_ and the oxidation of Fe^2+^ by O_2_ are also suppressed [69, 76].

**Fig. 7:**
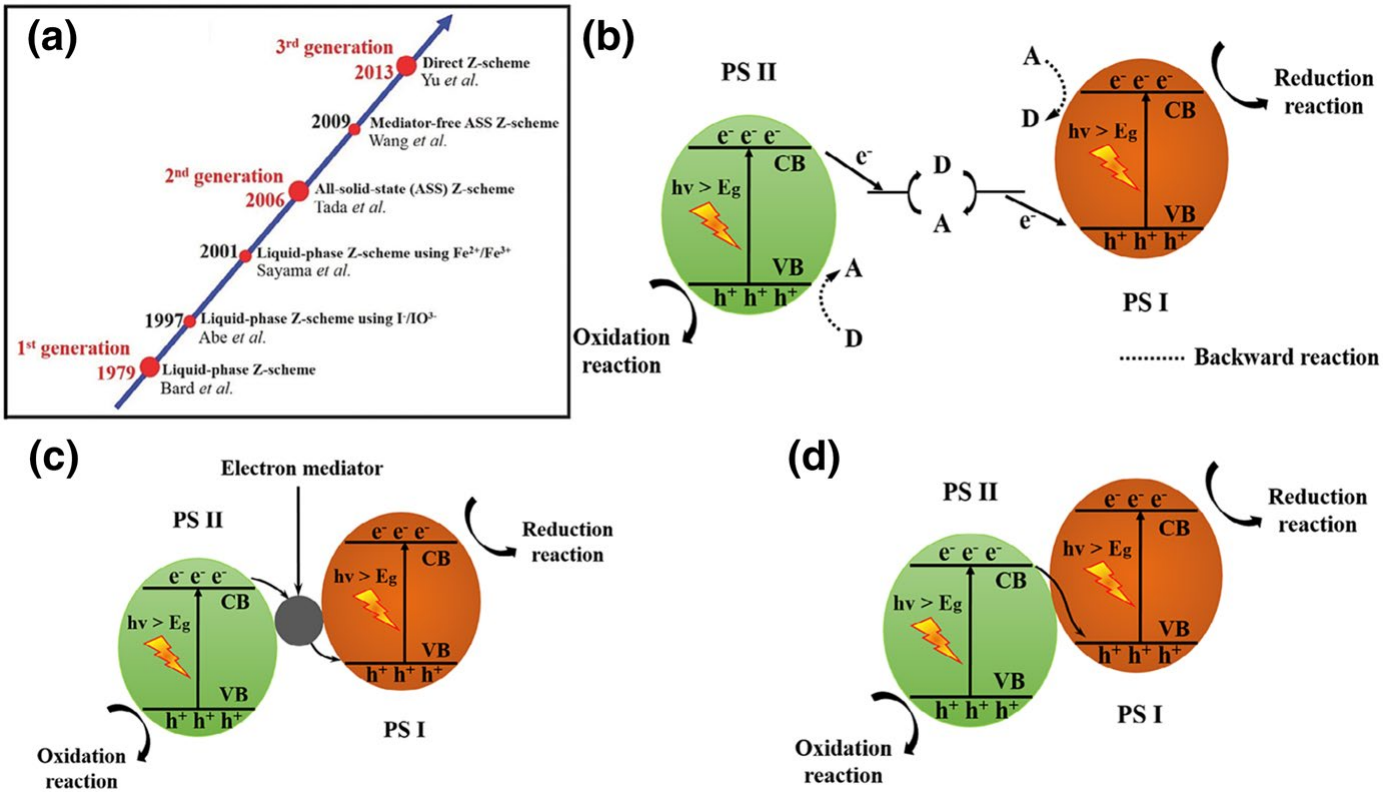
**a** The roadmap of the evolution of the Z-scheme photocatalytic system from the first generation to the third generation. **b** Schematic illustration of electron transfer in a traditional Z-scheme photocatalytic system (PS I-A/D-PS II), where A and D represent the electron acceptor and donor, respectively. **c** Schematic illustration of electron transfer in an all-solid-state Z-scheme photocatalytic system with an electron mediator (PS I–EM–PS II), where EM represents the electron mediator that provides an electron transport channel. **d** Schematic illustration of electron transfer in a direct Z-scheme photocatalytic system without any electron mediator (PS I–PS II). Reprinted with permission from [78] *©* 2020 Wiley-VCH

However, traditional Z-scheme photocatalysts have further drawbacks. Noteworthy are light-shielding effects by the redox mediator, slow charge carrier transfer rates limited by diffusion of ion pairs, and, as already mentioned, limitations because of pH sensitivity. Moreover, redox mediators are often unstable and tend to deactivate, which results in decreased reaction rates [77]. In addition, the photogenerated electrons in the CB of the photocatalysts I (PS I) and holes in the VB of photocatalysts II (PS II) with strong redox ability can also be consumed by the redox mediator (Fig. 7). This is called backward reaction and again has a negative impact on the photocatalysis [78, 79]. Several attempts have been made to overcome these drawbacks. The historical evolution of Z-scheme photocatalysts is shown in Fig. 7.

##### All-Solid-State Z-Schemes, Direct Z-Schemes

In 2006, Tada et al. proposed the concept of an all-solid-state Z-scheme heterojunction. The CdS-Au-TiO_2_ system used gold as a solid-state electron mediator [80]. As shown in Fig. 7c, the photocatalytic system consists of two semiconductors and a solid-state electron mediator between two semiconductors with an intimate contact. The intimate contact between the mediator and both semiconductors favours the interfacial charge carrier transfer [77, 78]. Commonly used solid-state electron mediators are noble metal (such as Au, Ag, Cu, Pt) shells/nanoparticles and graphene and carbon nanotubes. When the two semiconductors are excited by light irradiation, the photogenerated electrons in the CB of PS II can recombine with the photogenerated holes in the VB of PS I with the help of the electron mediator because of low contact resistances with both semiconductors [78, 81].

All-solid-state Z-schemes eliminate the aforementioned backward reactions and the long-term stability issues of redox mediators in traditional Z-schemes. Furthermore, it can be applied in liquid and gas phases because of the solid conductor [77, 78]. Light-shielding effects can be reduced compared to traditional Z-scheme photocatalytic systems, but effective light utilisation might be hindered by light absorption from the electron mediator. These conductors might also act as co-catalysts instead of charge-transfer shuttles. Therefore, intimate contact and carefully designed sandwiched structures are necessary, which is synthetically challenging. Additionally, the use of rare and noble metals limits practical applications of all-solid-state Z-schemes [77, 78, 82].

In 2013, a direct Z-scheme heterojunction (g-C_3_N_4_/TiO_2_) without using any electron mediator was reported by Yu et al. [83]. In contrast to the traditional Z-scheme system, the backward reactions were significantly suppressed because of the absence of redox mediators. The shielding effect caused by redox mediators/charge carrier mediators can also be reduced [77].

**Fig. 8:**
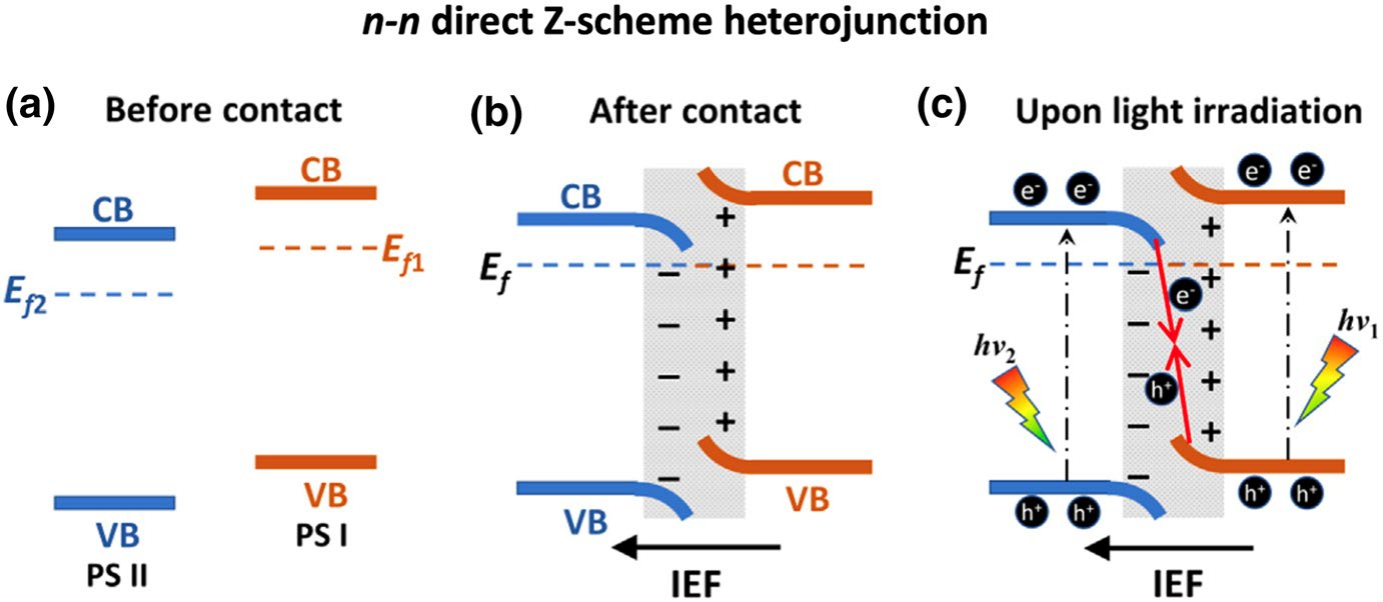
Schematic of *n–n* type heterojunction: **a** before contact, **b** after contact, and **c** formation of direct Z-scheme charge transfer upon light irradiation. E_f_ and IEF stand for Fermi level and internal electric field, respectively. Reprinted with permission from [82] © 2021 Elsevier

Analogous to the all-solid-state Z-scheme, the directly contacted semiconductors are both exited by light irradiation, and the photogenerated electrons in the CB of PS II can recombine with the holes in the VB of PS I. As a result, the photogenerated electrons in CB of PS I and photogenerated holes in the VB of PS II are spatially separated and maintain their strong redox capability [78]. A work function difference between the two semiconductors is a pre-requisite for the Z-scheme charge transfer mode. PS I must have higher CB and VB positions and a smaller work function (higher Fermi level) than PS II. When both semiconductors are in contact, an electron transfer from PS I to PS II takes place due to Fermi level equilibration (c.f. the *n*-*n* type direct Z-scheme in Fig. 8). Thus, the PS I side is positively charged, whereas the PS II side is negatively charged. Hence, an internal electric field (IEF) as well as band bending occurs. PS II energy band edges bend downwards because the accumulation of electrons and PS I energy band edges bends upwards because of decreased electron density [77, 82]. The IEF, the extra potential barrier induced by band bending, and Coulomb repulsion hinder photogenerated electrons from transferring from PS I CB to PS II CB. This also applies to the photogenerated holes in PS II VB [77].

**Fig. 9:**
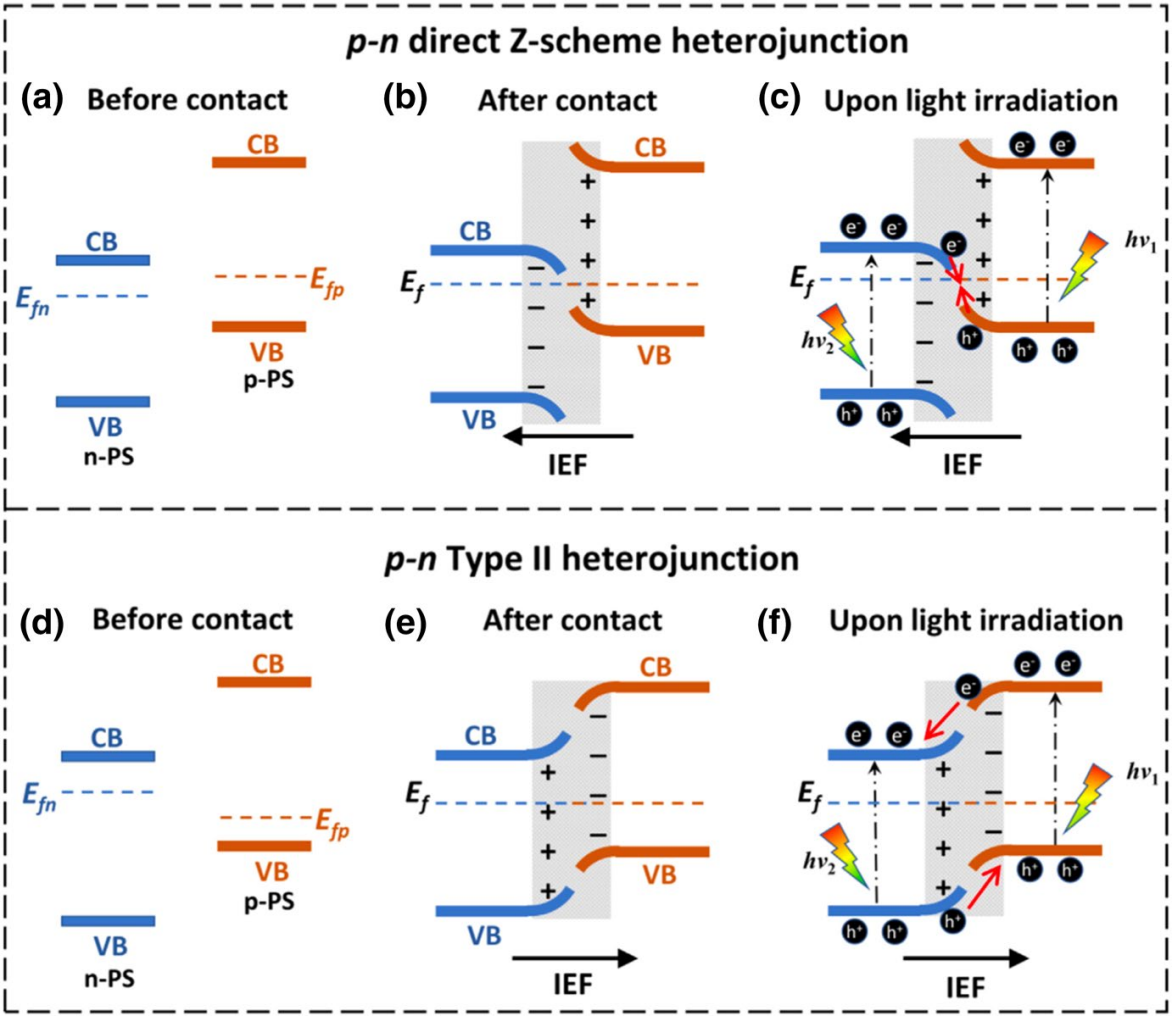
Schematic of **a**–**c** *p-n* direct Z-scheme heterojunction and **d**–**f** *p-n* type II heterojunction. Reprinted with permission from [82] © 2021 Elsevier

A similar process occurs in the construction of a *p*-*n* type direct Z-scheme. However, when the Fermi level of a *p*-type semiconductor (*p*-PS) is lower than that of an *n*-type semiconductor (*n*-PS), a type II heterojunction is formed. The energy band edges are bent in an inverted fashion at their interface (Fig. 9). Therefore, under light irradiation, photogenerated electrons move from *p*-PS to *n*-PS and holes in the opposite way driven by the IEF. The requirement for Fermi levels is easily met in *n-n* type heterojunctions but very hard to fulfil in *p-n* heterojunctions because of the huge band offset (Fig. 9). Hence, most direct Z-schemes are made of *n-n* heterojunctions [82].

Known *p-n* type direct Z-schemes are for example CuInS_2_–WO_3_, CuAl_2_O_4_–Bi_2_WO_6_, and Cu_2_O–TiO_2_ [82, 84–86].

The band alignment configuration in Z-scheme heterojunctions is similar to that of type II heterojunctions, but their charge transfer mechanisms are different. To investigate the exact charge transfer process, various methods can and should be used. Several methods have been reported: self-confirmation by photocatalytic reaction products and radical species; selective photodeposition of a noble metal; in situ irradiated x-ray photoelectron spectroscopy (XPS) analysis; surface photovoltage (SPV) technique; time-resolved diffuse reflectance (TDR) spectroscopic analysis [78]. Sometimes, photo-corrosion effects are reduced, which can be a hint for a Z-scheme mechanism (e.g. in composite materials containing CdS) [87].

The investigation of charge transfer processes is essential to describe the photocatalytic activity in a given system and to improve photocatalysts in the future. As mentioned before, heterojunctions and Z-schemes can show undesired charge transfers and backward reactions. We would like to mention that the term S-scheme (step scheme) was also introduced and is mainly synonymously used with direct Z-schemes [88–90]. In this article, the term direct Z-scheme instead of S-scheme is used.

##### Facets and Regulation of Charge Flow Direction

**Fig. 10:**
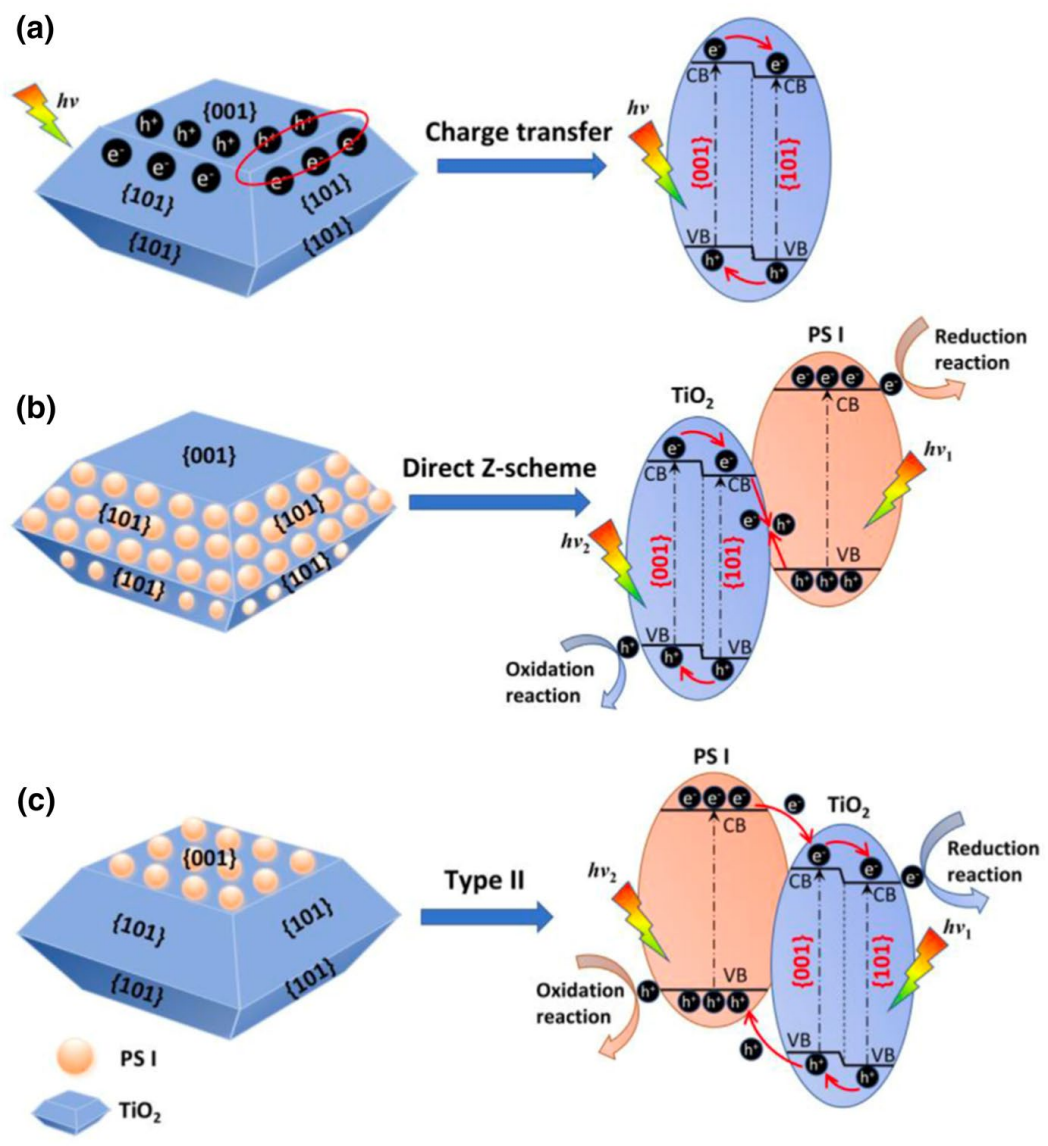
Charge transfer **a** at the edge of a single TiO_2_ nanocrystal, **b** in a direct Z-scheme, and **c** in a type II TiO_2_-based heterojunctions induced by facet design. Reprinted with permission from [82] © 2021 Elsevier

Facet design has been discussed many times for various photocatalysts [91, 92]. Direct Z-schemes can also be constructed and improved through facet design. Here, charge flow directions and the recombination of photogenerated charges at interfaces can be regulated [82]. The co-exposed {0 0 1} and {1 0 1} facets of anatase TiO_2_, for example, would form a “surface heterojunction” as shown in Fig. 10a. Due to different band structures and band edge positions, photogenerated electrons and holes would transfer to {1 0 1} and {0 0 1} facets, respectively [82, 93].

Anchoring a suitable semiconductor with a smaller work function (higher Fermi level) on the {1 0 0} facet of TiO_2_, would lead to the formation of a Z-scheme (Fig. 10b). In contrast, if said semiconductor is deposited on {0 0 1} facets, a type II heterojunction will be formed instead [82]. In 2015, a facet-induced direct Z-scheme between TiO_2_ {1 0 1} and g-C_3_N_4_ was realised by Huang et al. [94]*.*

Jiang et al. used chemical deposition and photodeposition to regulate the electron flow direction in g-C_3_N_4_/CdS composite materials [95]. In the photodeposition case, a type II heterojunction was constructed because CdS was selectively deposited at the electron transfer site of g-C_3_N_4_, resulting in a photogenerated electron transfer from g-C_3_N_4_ to CdS. Using a chemical deposition technique, CdS was randomly deposited onto g-C_3_N_4,_ which resulted in a Z-scheme charge transfer mechanism [95].

**Fig. 11:**
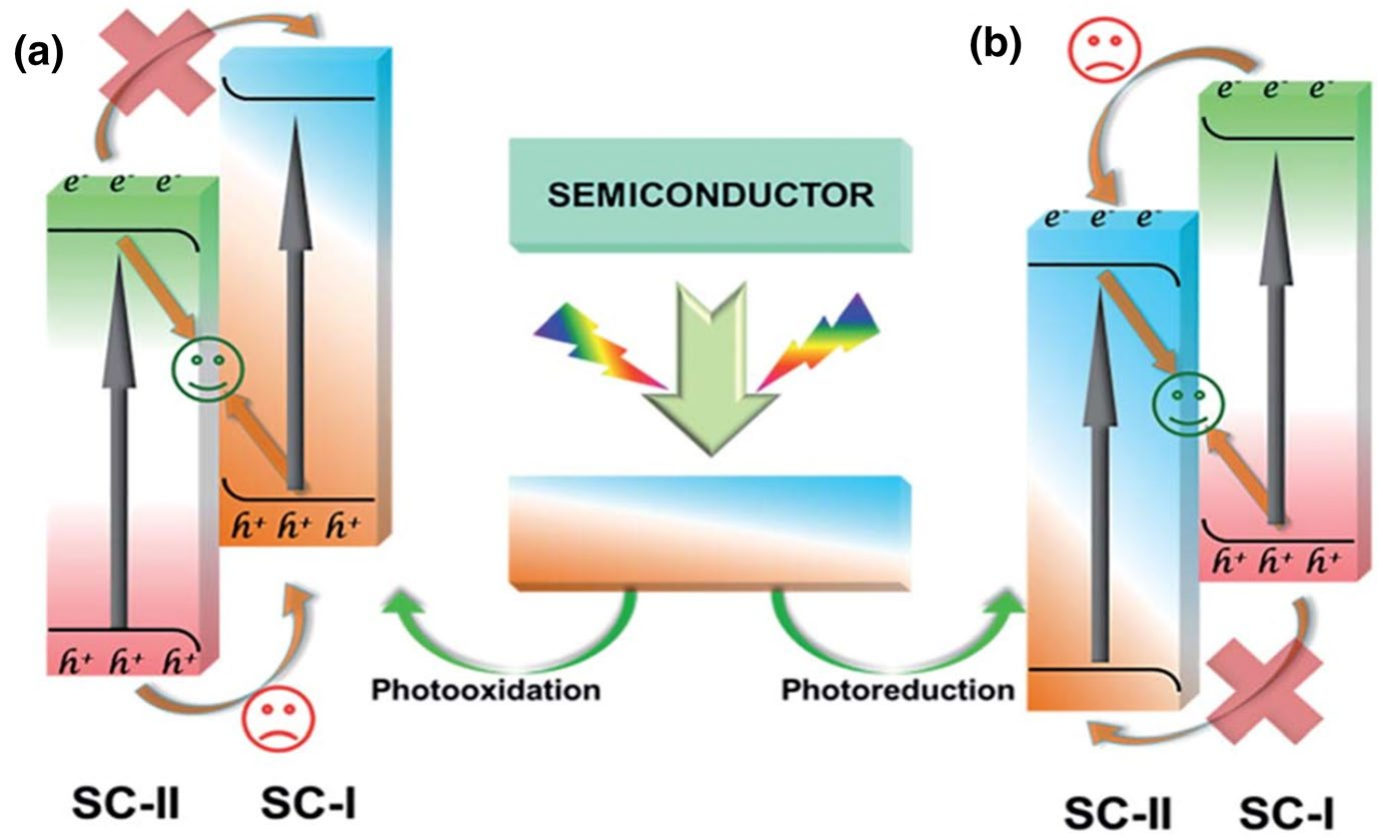
The construction routes of direct Z-scheme photocatalysts through photodeposition: **a** photodeposition of SC-II (e.g. Fe_2_O_3_) on SC-I (e.g. g-C_3_N_4_) through photooxidation; **b** photodeposition of SC-I (e.g. CdS) on SC-II (e.g. TiO_2_) through photoreduction (SC = semiconductor). Reprinted with permission from [96] © 2019 Royal Society of Chemistry

The same group showed in 2019 that deliberate construction of direct Z-scheme photocatalysts was possible by photodeposition [96]. Two routes to construct direct Z-schemes were proposed via photooxidation and photoreduction (Fig. 11).

When g-C_3_N_4_ was selected as SC-I, Fe_2_O_3_ as SC-II was selectively deposited on hole-rich sites of g-C_3_N_4_ through photooxidation (Fig. 11a). When CdS was selected as SC-I, it was selectively deposited on electron rich sites of SC-II (TiO_2_) (Fig. 11b) [96]. In the future, further investigations might allow to transfer this approach to other materials. A foundation would be extensive investigations of electron- and hole-rich sites and the deliberate construction of these sites for different materials.

##### Dual Direct Z-Schemes

Direct Z-schemes can also be built from a combination of three semiconductors. These photocatalysts are usually called “dual Z-schemes” or “ternary Z-schemes” [82]. One advantage of these systems is the better spatial separation of reduction and oxidation reactions which occurs on nonadjacent semiconductors. As a result, recombination rates can be reduced or, in other words, the lifetime of photogenerated charge carriers can be prolonged [82, 97, 98]. Furthermore, a broader light absorption spectrum is achievable [98].

**Fig. 12:**
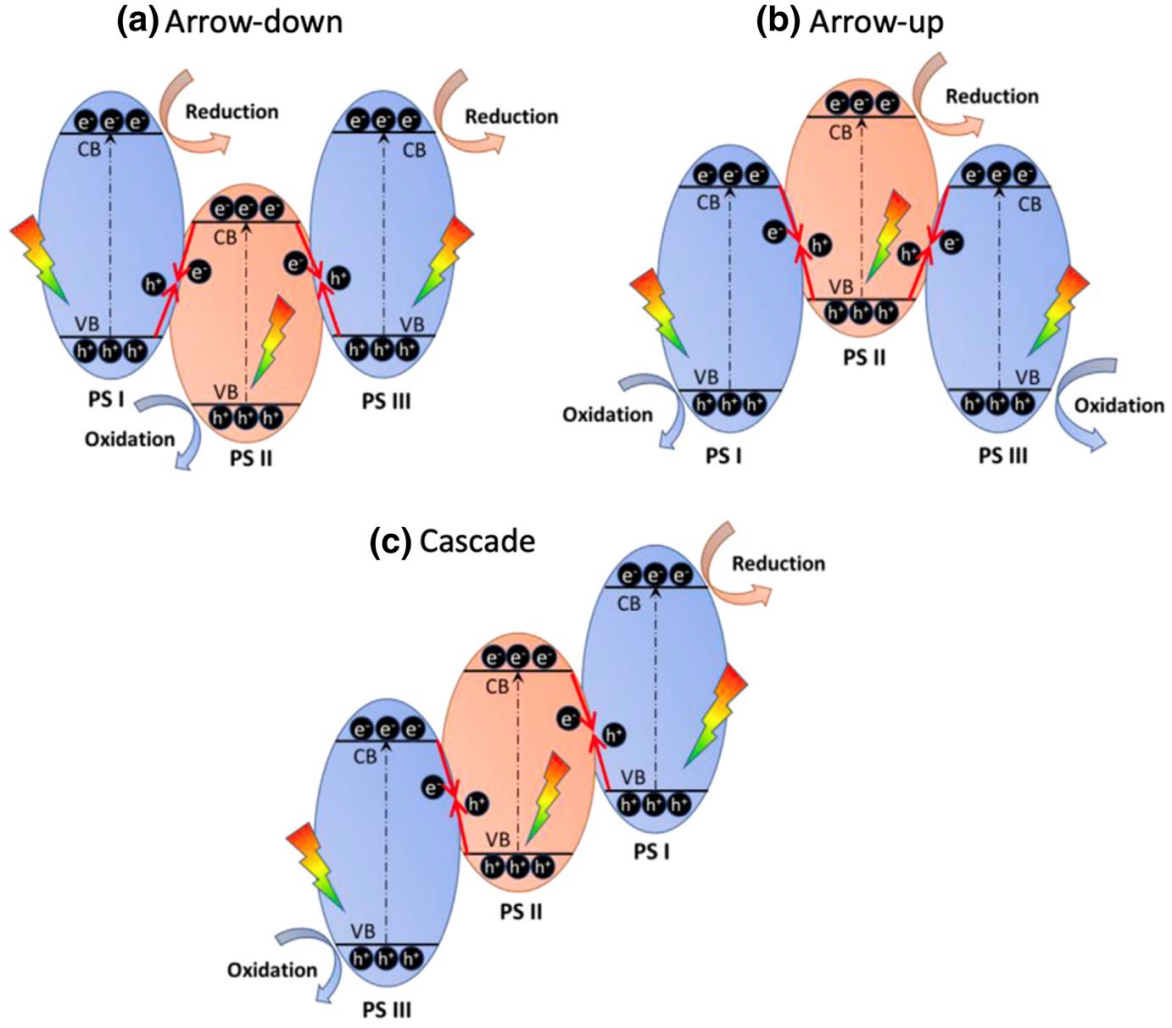
Band alignments in three types of dual direct Z-schemes: **a** arrow-down, **b** arrow-up, and **c** cascade. Reprinted with permission from [82] © 2021 Elsevier

Li et al. divide dual direct Z-schemes into three categories (Fig. 12). According to the shape formed by the position of conduction bands and valence bands relative to each other, these systems are called arrow-down, arrow-up, and cascade dual Z-schemes [82].

In an arrow-down system, the electrons in the CB of PS II combine with holes in the VBs of PS I and PS III. Therefore, the electrons at the CBs of PS I and PS III participate in the reduction reaction while the holes in the VB of PS II participate in the oxidation reaction. In an arrow-up photocatalyst, the band positions are inverted compared to the former described system. Therefore, the reduction takes place at PS II while oxidation takes place at PS I and PS III. In a cascade dual Z-scheme, the electrons in the CB and holes in the VB of PS II combine with the holes in the VB of PS I and electrons in the CB of PS III, respectively [82].

All three types of dual Z-schemes have been realised in the past few years. An arrow-down dual Z-scheme was demonstrated by Xue et al. in the form of a g-C_3_N_4_/Bi_2_WO_6_/AgI combination [99]. An arrow-up dual Z-scheme was reported by Zheng et al. for a Co_3_O_4_@CoO/g-C_3_N_4_ combination [100]. In 2019, Wang et al. reported a g-C_3_N_4_/Zn_2_SnO_4_N/ZnO cascade dual Z-scheme [97].

**Fig. 13:**
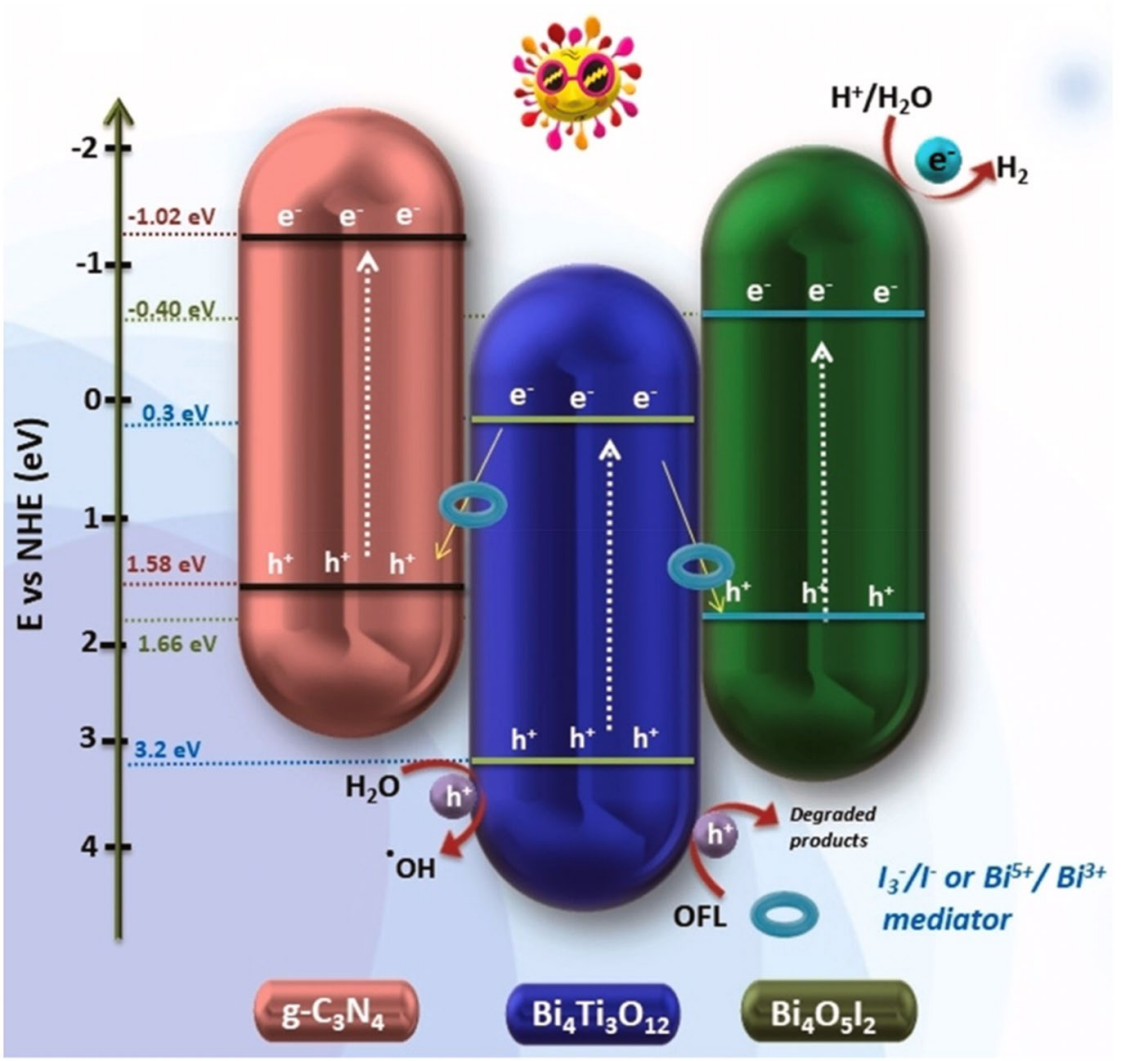
Photocatalytic mechanism for H_2_ evolution and pollutant degradation by dual Z-scheme mechanism. Reprinted with permission from [101] © 2021 Elsevier

Unfortunately, none of these three systems were tested for water splitting or H_2_ evolution reactions. In the past 3–4 years, more and more dual direct Z-schemes have been reported aiming at H_2_ evolution. Recently, Kumar et al. reported a g-C_3_N_4_/Bi_4_Ti_3_O_12_/Bi_4_O_5_I_2_ arrow-down dual Z-scheme for photocatalytic antibiotic degradation and H_2_ production (Fig. 13) [101].

XPS measurements confirmed that the double Z-scheme mechanism is facilitated or mediated by in-built redox mediators Bi^5+^/Bi^3+^ and I_3_^–^/I^–^ or IO_3_^–^/I^–^. Thus, a complicated dual direct Z-scheme assisted by redox mediators was constructed. Aside from antibiotic degradation, the system was also tested for photocatalytic H_2_ evolution in pure water and in solutions containing triethanolamine (TEOA, 6.7·10^–5^ M) reaching 24.12 mmol g^−1^ h^−1^ and 69 mmol g^−1^ h^−1^, respectively (Table 1 in the Appendix).

Another ternary structure for photocatalytic H_2_ evolution reported by Dong et al. used a ZnO/ZnS/g-C_3_N_4_ cascade dual Z-scheme. The composite displayed specific surface areas of 76.6 m^−2^ g^−1^ and reached a H_2_ evolution rate of 0.3 mmol g^−1^ h^−1^ using sacrificial agents (Na_2_S and Na_2_SO_3_) but no co-catalysts [102].

In summary, dual direct Z-schemes are a recent trend and further reports focusing on H_2_ evolution reactions and water splitting are expected for the future. Nevertheless, these structures suffer from the limitation of material availability to achieve suitable band alignments [82]. Also, complex synthesis protocols and even more challenging charge transfer analyses are necessary to optimise and understand these systems.

##### Z-Schemes with MOFs and COFs

**Fig. 14:**
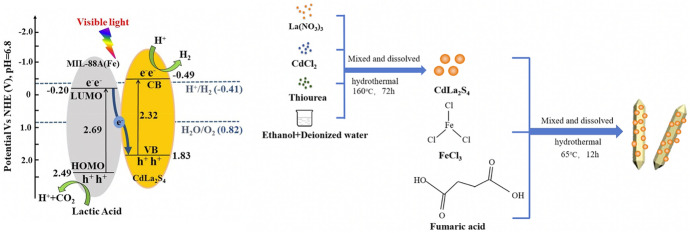
Band alignments in the CdLa_2_S_4_/MIL-88A(Fe) Z-scheme (left) and synthesis process of the composite (right). Reprinted with permission from [104] © 2019 Elsevier

Metal organic frameworks (MOFs) are one-, two-, or three-dimensional coordination networks consisting of metal ions/clusters linked together by organic linker molecules. MOFs have received growing attention in the construction of direct Z-schemes. Advantages like tuneable composition and light-harvesting property, high surface areas, and controllable pore sizes make MOFs attractive in applications of photocatalysis [82, 103]. The visible light photocatalytic performance of pure MOFs is quite poor. However, in direct Z-schemes significant improvements have been reported [82, 104–106]. Various morphologies are easily achievable, which enables the use of 3D, 2D, and 1D structures in direct Z-schemes. Chen et al. constructed a direct Z-scheme by anchoring CdLa_2_S_4_ nanoparticles on 1D MIL-88A(Fe) micro-rods (Fig. 14). The nanocomposite obtained high H_2_ evolution rates of up to 7.678 mmol g^−1^ h^−1^ using a 20 wt% content of MIL-88A(Fe). Moreover, the CdLa_2_S_4_/MIL-88A(Fe) composite exhibited high durability during the photocatalytic H_2_ evolution [104].

The high photocatalytic activity mainly contributed to the formed Z-scheme structure [104].

**Fig. 15:**
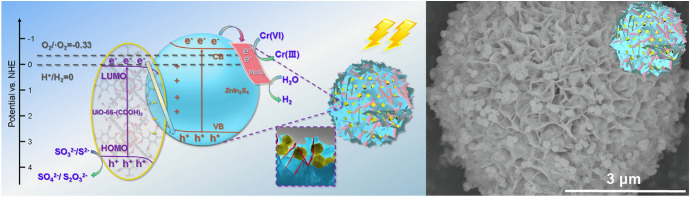
Proposed photocatalytic Z-scheme mechanism (left) and field-emission scanning electron microscope (FESEM) image of the UiO-66-(COOH)_2_/ZnIn_2_S_4_ (yellow/blue) photocatalyst decorated with MoS_2_ co-catalyst (red) (right). Reprinted with permission from [106] © 2019 Elsevier

In 2019, a 3D hierarchical structure of a UiO-66-(COOH)_2_/ZnIn_2_S_4_ Z-scheme was reported [106]. Here, UiO-66-(COOH)_2_ nanoparticles were decorated in the interweaving petal nanosheets of ZnIn_2_S_4_ microspheres, while co-catalyst MoS_2_ nanosheets were folded at the edge of these interweaving petals as shown in Fig. 15.

The optimised photocatalyst showed high H_2_ evolution rates of 18.794 mmol g^−1^ h^−1^, which was about 15.3 times higher than the H_2_ evolution rate of pure ZnIn_2_S_4_.

Overall, only a small group of MOFs are suitable for H_2_ evolving or water splitting photocatalytic applications since most MOFs are known to become unstable if exposed to moisture/water. However, > 40 MOFs are known to show enhanced water stability (amongst others MIL-100, MIL-101, UiO-66, Zn-DMOF, DUT-67) [107]. Considering possible morphologies and composite materials containing MOFs, there still seems to be potential for stable and low-cost direct Z-schemes in the future.

Covalent organic frameworks (COFs) are crystalline covalent polymers with a high surface area. These porous materials with tuneable topology and functionalities have gathered significant interest among researchers in the field of photocatalysis [108, 109]. The moderate band gap and the presence of extended π-conjugated electronic networks enable visible-light responsive ability and possibilities for bandgap engineering. Therefore, COFs and COF-linked hybrid materials are used as photocatalysts for H_2_ production. Due to the stability of covalent bonds compared to coordinate bonds in MOFs, COFs are known to exhibit high stability in various solvents even under harsh acidic, basic, oxidative, or reductive conditions. However, a majority of reported photocatalytic COFs are based on imine, hydrazone, or azine linkages and are considered unstable for extended photocatalysis in water [108]. Currently, only a few COF materials have been investigated for photocatalytic H_2_ evolution or water splitting. Nonetheless, there has been considerable progress regarding stability and photocatalytic activity in the past few years. One example is sulfone-containing COFs reported in 2018 by Wang et al. [110]. These covalent organic frameworks are based on a benzobis(benzothiophene sulfone) moiety and are stable for at least 50 h in water under visible light irradiation. Using ascorbic acid (0.1 M) as a sacrificial electron donor and Pt as a co-catalyst (8 wt%), H_2_ evolution rates of up to 10.1 mmol g^−1^ h^−1^ were obtained (300 W Xe light source). Without the addition of co-catalyst, still significant rates of up to 1.32 mmol g^−1^ h^−1^ were reported [110]. Their internal pore structure could be decorated with nanoparticles/quantum dots, making these COFs a platform for developing hybrid photocatalysts.

**Fig. 16:**
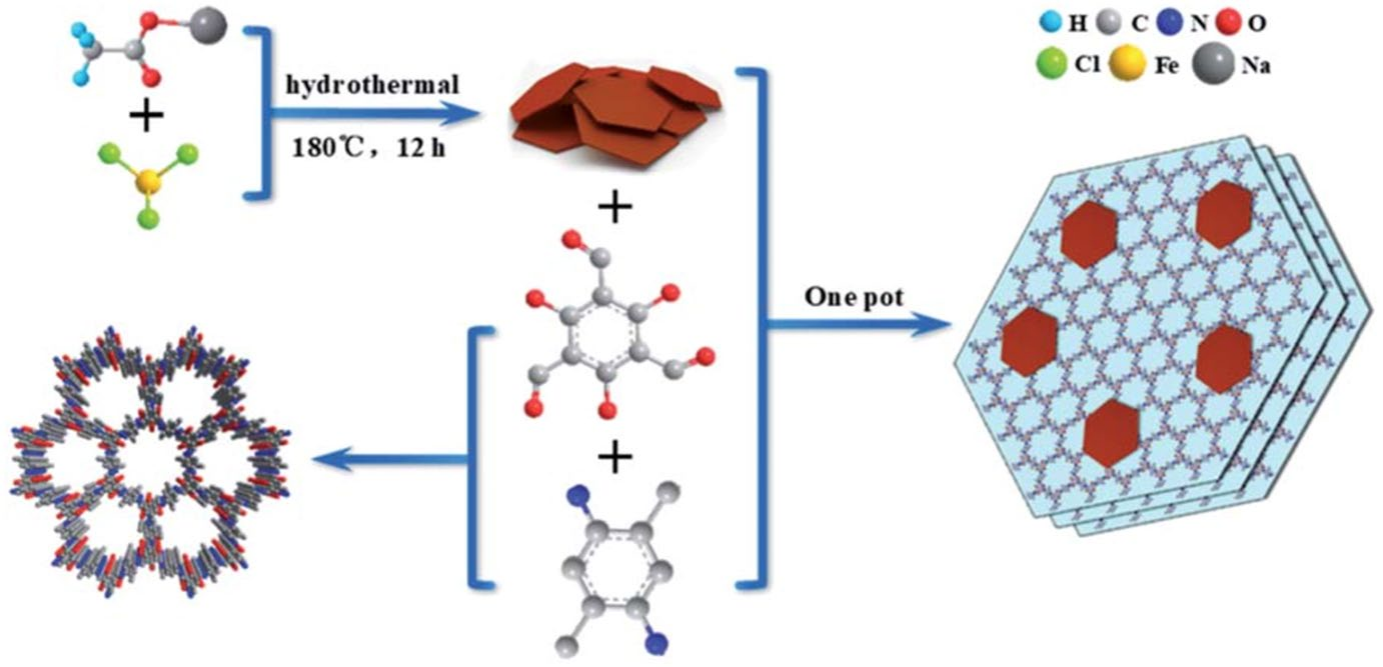
Schematic illustration of the synthesis of an $\alpha$-Fe_2_O_3_/TpPa-2-COF hybrid material. Reprinted with permission from [111] © 2020 Royal Society of Chemistry

In 2020, a COF-based noble-metal-free Z-scheme (TpPa-2-COF/$\alpha$-Fe_2_O_3_) was reported for the first time [111]. The transfer of photogenerated electrons from the CB of $\alpha$-Fe_2_O_3_ to the VB of the TpPa-2-COF was confirmed, and the effectiveness of the Z-scheme structure attributed to the tight integration between the metal oxide and the COF. As illustrated in Fig. 16, the hybrid material was synthesised by a simple one-pot synthesis using $\alpha$-Fe_2_O_3_, 1,3,5-triformylphloroglucinol and 2,5-dimethyl-*p*-phenylenediamine.

The co-catalyst free Z-scheme showed H_2_ evolution rates of up to 3.77 mmol g^−1^ h^−1^, which is about 53 times higher than that of the parent TpPa-2-COF und the same conditions and even better than that with a Pt (2 wt%) co-catalyst [111].

Compared to MOFs, COFs are still “young”, but their cheap synthesis, tuneable pores, and other characteristics like visible-light absorption make them attractive for the application as photocatalysts. Hybrids with metal-oxides have already demonstrated significant performance improvements [82]. Hybrid materials based on already optimised COFs from recent publications might lead to even higher photocatalytic activity.

##### Coupling Surface Plasmon Resonance (SPR) Effect into Direct Z-Scheme Systems

Another strategy to improve photocatalytic activity is coupling surface plasmon resonance (SPR) into direct Z-schemes. SPR is the collective oscillation of electrons that are in resonance with the oscillating electric field of incident light [112]. Resonances near the visible or near IR range can enhance the light absorption ability of the photocatalytic system. In addition, SPR-induced hot plasmon electrons or SPR-enhanced localised electromagnetic fields can boost the charge separation near the interfaces between the SPR material and the semiconductor [82, 113, 114]. Well-known materials are noble metals (Ag and Au) and heavily doped semiconductors (H_x_WO_3_, W_18_O_49_ and BiO_2-x_) [82].

Among these materials, noble metals are most commonly used for application in photocatalysis. The Schottky junction supplies an internal electric field between the noble metal and the semiconductor, which improves the interfacial charge transfer. Localised surface plasmon resonances (LSPR) can enhance solar light absorption. Both features can lead to significant enhancement of the photo-reactivity [82, 113]. This was described, for example, in TiO_2_/Au/WO_3_ or TiO_2_/Ag/Cu_2_O Z-schemes for H_2_ generation [115, 116]. Other examples or Z-scheme couples with LSPR for photocatalytic H_2_ generation include TiO_2_/Pt/WO_3_ [117], TiO_2_/Au/CdS [118], C_3_N_4_/Au/TiO_2_ [119], C_3_N_4_/Ag/NiTiO_3_ [120], C_3_N_4_/Ag/SnS_2_ [121], SrTiO_3_/C_3_N_4_/Ag/Fe_3_O_4_ [122], and C_3_N_4_/Ag/CdS [123]. In contrast, for a Ag/C_3_N_4_/TiO_2_ system, a SPR-enhanced heterojunction was reported, with Ag not acting as electron mediator simultaneously [124].

Xie et al. combined modified C_3_N_4_ (NCN-CN_x_) with gold nanoparticles and CuInS_2_ to achieve photocatalytic hydrogen evolution from water/triethanolamine, reaching 4.28 mmol h^−1^ g^−1^ at wavelengths > 550 nm [125]. Interestingly, surface photovoltage spectroscopy was additionally used to confirm the SPR effect and the synergistic effects of SPR and Z-scheme mechanism.

Modulating the stoichiometric ratio of certain semiconductors is also used to improve light absorption abilities since it can increase the free-charge density and lead to LSPR arising from collective oscillations of the excess free-charges on the semiconductor surface [126]. Blue W_18_O_49_ is a nonstoichiometric tungsten oxide with a bandgap of 3.0 eV and exhibits LSPR absorption in both the vis and NIR region because of abundant oxygen vacancies on its surface. Zhang et al. used this material to synthesise a g-C_3_N_4_/W_18_O_49_ plasmonic Z-scheme photocatalyst, which could harvest photon energies spanning from the UV to the near IR region [126]. A wavelength range of 450–900 nm electrons near the Fermi level of W_18_O_49_ reached the high-energy surface plasmon state and became plasmonic “hot electrons”, which then could partly transfer to the conduction band of g-C_3_N_4_. The system reached H_2_ evolution rates of up to 3.04 mmol g^−1^ h^−1^ when triethanolamine (15 vol%) was used as sacrificial agent.

Although considerable progress has been made in plasmonic metal-based and nonmetal-based photocatalysts, challenges remain such as low utilisation of hot carriers, uncovered mechanisms of hot carrier-driven reactions, and the exploration of novel (nonmetallic) plasmonic materials. Therefore, the full potential of plasmonic materials for efficient photocatalytic systems is yet to be realised [127].

##### Upconversion Photoluminescence (UCPL) Materials in Z-Schemes

Besides the (L)SPR materials, most direct Z-scheme photocatalysts can only utilise the light energy in the UV and visible region. These materials' inability to utilise light from the near IR (NIR) region is an impediment in the progressive photocatalysis research since NIR light constitutes the maximum portion of solar energy (approximately 50%) [128]. Therefore, efficient full-spectrum-activated (UV–Vis–NIR) photocatalysts have gained a lot of attention. An attractive strategy is to integrate non-linear UCPL materials in direct Z-scheme systems [82, 129].

**Fig. 17:**
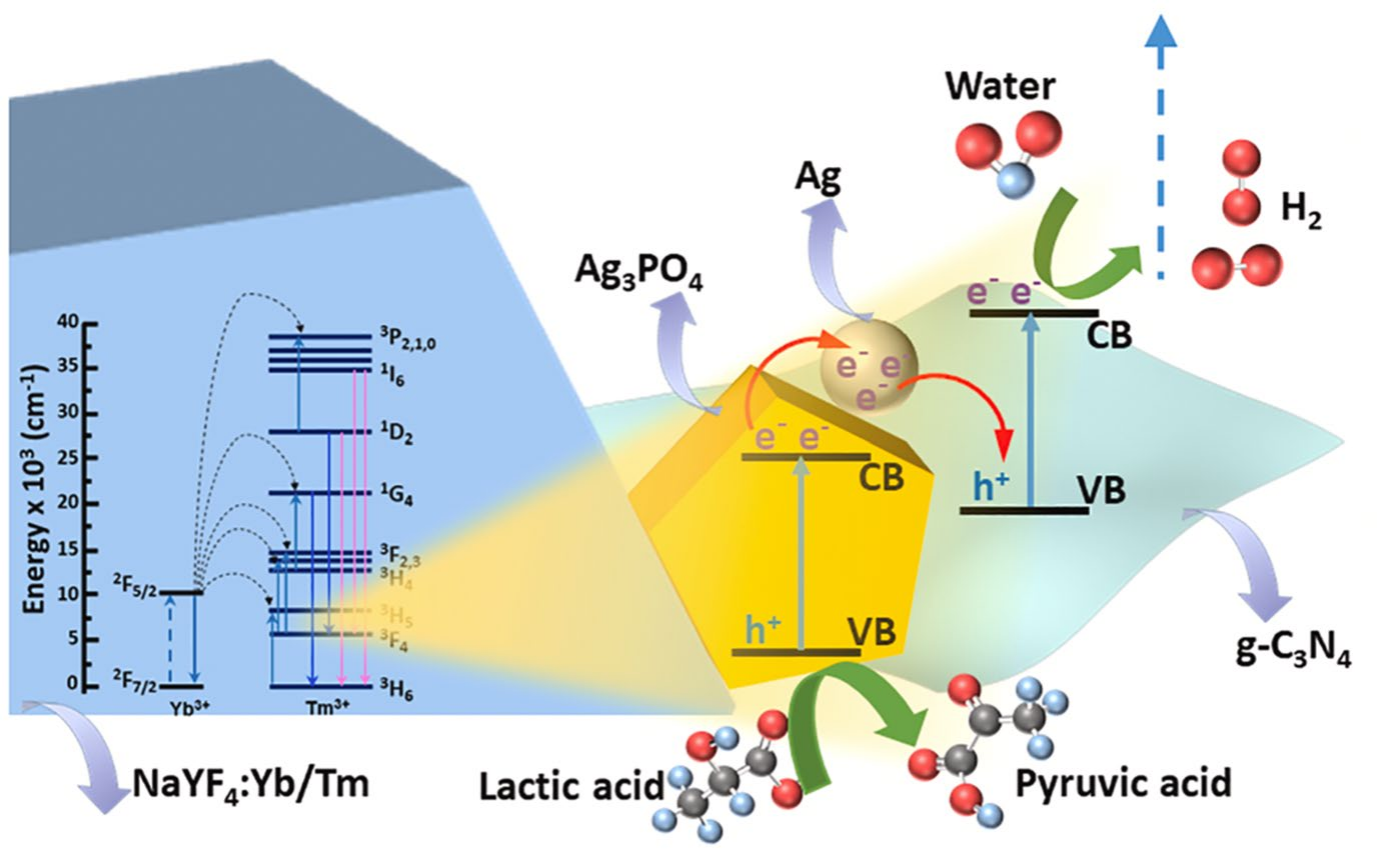
Schematic representation of the photocatalytic H_2_ evolution mechanism of g-C_3_N_4_/Ag_3_PO_4_ Z-scheme photocatalyst under the sunlight illumination using Yb/Tm-doped NaYF_4_ as an UCPL material and lactic acid as a sacrificial agent. Reprinted with permission from [128] © 2021 Elsevier

UCPL materials exhibit a multi-photon-assisted anti-Stokes excitation mechanism, which enables the conversion of low energy (NIR) photons into radiation of UV and visible light [130]. Rare-earth-based lanthanide-doped materials, such as Yb^3+^- and Er^3+^-doped NaYF_4_, are popular UCPL substances [82]. Unfortunately, rare earth-doped upconversion materials generally suffer from low upconversion efficiency due to small absorption cross sections and low energy transfer efficiencies [131]. In doped NaYF_4_, the upconversion characteristics entirely depend on the selection of dopants [132]. Yb/Tm-doped NaYF_4_, for example, exhibits several UV–visible upconversion emission peaks that fall under the absorption range of semiconductor photocatalysts. Recently, this material has been used by Murali et al. for a NIR-activated g-C_3_N_4_/Ag_3_PO_4_ Z-scheme [128]. The mechanism behind the NIR-light utilised photocatalytic H_2_ production is shown in Fig. 17.

The NIR photons in solar irradiation excite Yb^3+^ ions in the NaYF_4_ host from the ground state (^2^F_7/2_) to the excited state (^2^F_5/2_). These excited Yb^3+^ ions act as sensitisers and populate higher energy levels of Tm^3+^ ions. Non-radiative relaxation of Tm^3+^ ions populates several other of its energy levels. The energy is then transferred to the g-C_3_N_4_/Ag_3_PO_4_ via Förster resonance energy transfer (FRET). The absorption of additional energy by the UV–visible g-C_3_N_4_/Ag_3_PO_4_ Z-scheme spurs the number of free charges. In this particular system, Ag^0^ comprised in Ag_3_PO_4_ additionally acts as a channel between both semiconductors [128].

Yb/Tm-doped NaYF_4_/Ag_3_PO_4_ was also combined with black phosphorus for another Z-scheme/UCPL heterostructure [133]. Interestingly, a laser source (980 nm) was used as light source to prove the NIR upconversion; 0.146 mmol g^−1^ h^−1^ H_2_ evolution from aqueous glycerol solution (50 vol%) could be achieved, with 0.077% apparent quantum efficiency at 980 nm.

TiO_2_ was also used in combination with other semiconductors and UCPL materials for photocatalytic H_2_ evolution. Together with g-C_3_N_4_ and carbon quantum dots, photocatalytic water splitting was achieved in a 2:1 ratio at a maximum H_2_ evolution rate of 6.497 µmol h^−1^ g^−1^ under 300 W Xe illumination (> 400 nm) [134]. Therein, the carbon quantum dots are supposed to have a dual function as upconversion material and catalyst for H_2_O_2_ decomposition, therefore facilitating oxygen evolution and water splitting.

Iron-doped TiO_2_ combined with vanadium-doped Ta_2_O_5_ and Er^3+^:YAlO_3_ as UCPL were applied for photocatalytic H_2_ generation from aqueous methanol solutions, including decoration with gold nanoparticles [135]. Er^3+^:YAlO_3_ was used as UCPL agent from visible-light to ultraviolet-light to be able to excite the wide band gap oxides with visible light, and a Z-scheme was established by the dopants resulting in a TiO_2_-Fe^3+^/V^5+^-Ta_2_O_5_ redox cycle system. By irradiation with a 300-W Xe lamp and a cut-off filter, H_2_ evolution activity of approximately 32 µmol h^−1^ g^−1^ was shown in visible light.

These examples show that the combination of UCPL materials and Z-schemes or heterojunctions is another promising method to enhance the sunlight absorption of semiconductor photocatalyst systems for H_2_ generation and water splitting. More efforts are however needed to optimize the FRET to the semiconductors to improve the overall solar-to-H_2_ efficiency.

##### 2D/2D Z-Schemes

Among the various developed photocatalysts, 2D materials are receiving increasing attention because of their unique physical and chemical properties [136]. Compared with bulk photocatalysts, 2D photocatalysts have a higher specific surface area due to their planar structure and ultralow thickness. Additionally, more surface atoms can provide more adsorption and active sites for photocatalytic reactions [78]. Constructing 2D/2D Z-scheme heterojunctions can combine the respective advantages of 2D materials and Z-scheme systems.

Zhu et al. fabricated a 2D/2D BP/BiVO_4_ Z-scheme (BP = black phosphorus) and realised overall water splitting [137, 138]. Thin BP and BiVO_4_ were hybridised by electrostatic interactions. In both photocatalytic H_2_ and O_2_ production processes, the 2D/2D Z-scheme exhibited better photocatalytic activities than BP and BiVO_4_ alone. H_2_ and O_2_ production rates of 0.16 mmol g^−1^ h^−1^ and 0.102 mmol g^−1^ h^−1^, respectively, and an apparent quantum efficiency of 0.89% were realised [137, 138].

**Fig. 18:**
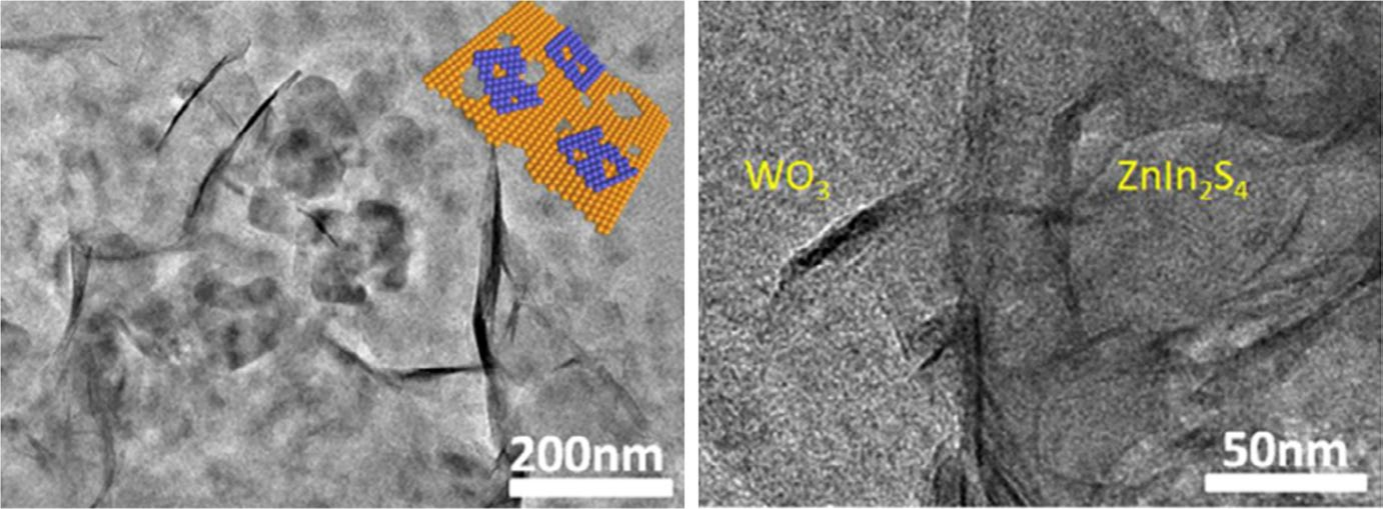
TEM images of the 2D/2D ZnIn_2_S_4_/WO_3_(10 wt%) Z-scheme. Reprinted with permission from [139] © 2019 Elsevier

Li et al. prepared a Janus sulphur vacancy-rich ZnIn_2_S_4_/WO_3_ Z-scheme [139]. The Janus bilayer was fabricated via electrostatic self-assembly by adding WO_3_ nanosheets into the precursor of ZnIn_2_S_4_. TEM images of the 2D/2D ZnIn_2_S_4_/WO_3_ (10 wt%) composite are shown in Fig. 18.

Vacancy-rich ZnIn_2_S_4_ was chosen because the sulphur vacancies significantly promoted H_2_ production due to optimised charge separation and light utilisation. The Z-scheme photocatalyst obtained H_2_ evolution rates of up to 7.81 mmol g^−1^ h^−1^, which was about 20.8 and 1.69 times higher than those of ZnIn_2_S_4_ and Pt-ZnIn_2_S_4_. In addition, the loading of NiS quantum dots as co-catalyst could further improve the photocatalytic H_2_ production, which reached 11.09 mmol g^−1^ h^−1^. This is the highest photocatalytic activity toward visible-light-driven H_2_ evolution among the family of ZnIn_2_S_4_ materials reported so far.

In addition to the previous examples, 2D/2D Z-schemes were also reported for g-C_3_N_4_/Fe_2_O_3_ [140], g-C_3_N_4_/WO_3_ [141], g-C_3_N_4_/BiVO_4_ [142], Zn_0.67_Cd_0.33_S/Cu_2_S [143], Zn_x_Cd_1-x_S/ZnO [144], g-C_3_N_4_/La_2_Ti_2_O_7_ [145], and several other composite materials. Some of these 2D/2D Z-schemes are listed in Table 1 in the appendix.

More than 3000 papers on 2D/2D Z-scheme photocatalyst have been published, more than 700 of them in 2021 alone (data from SciFinder^n^). Research on 2D/2D composites is therefore a major trend in the field of Z-scheme photocatalysis, which cannot be fully covered in this review. Interested readers are referred to other excellent reviews [78, 146–149].

## Comparison, Trends, and Conclusion

Considering heterojunctions and Z-schemes reported in the last 5 years, high H_2_ evolution rates were achieved, for example, for CdLa_2_S_4_/MIL-88(Fe) (7.678 mmol g^−1^ h^−1^) [104], g-C_3_N_4_/CdS (10.89 mmol g^−1^ h^−1^) [150], g-C_3_N_4_/Ag_3_PO_4_ (23.56 mmol g^−1^ h^−1^) [128], CoTiO_3_/UiO-66 (26.545 mmol g^−1^ h^−1^) [151], CdS/(Au/)TiO_2_ (47.6 mmol g^−1^ h^−1^) [118], CdS/BiVO_4_ (23 and 57 mmol g ^1^ h^−1^) [87, 152], g-C_3_N_4_/Bi_4_Ti_3_O_12_/Bi_4_O_5_I_2_ (69 mmol g^−1^ h^−1^) [101], CdS/ZnO (98.82 mmol g^−1^ h^−1^) [153], and several other photocatalytic systems (Table 1 in the Appendix).

Unfortunately, these values are not comparable because of differences in measurement setups, light sources, intensity, filters, co-catalysts, and several other factors. H_2_ evolution rates from > 50 publications have been evaluated and are listed in Table 1 in the appendix. Nevertheless, we hope that the readers of this article find it useful to compare some parameters used for hydrogen production in literature recently. Some of the production rates are very high and could also be a result of normalising the given rate in the publication to the used catalyst mass. Furthermore, high rates were often reported when low catalyst weights (< 20 mg) were used [139, 152, 153]. This may indicate that rather small setups were used or that weighing errors can lead to incorrect production rates. In some cases, no production rates were given, and we had to estimate hydrogen production rates from graphical depictions [128, 154, 155]. It is moreover well known that in photocatalysis the observed rates should not be normalized to the photocatalyst mass because of scattering and shadowing effects leading to non-linear correlation of photocatalyst mass and activity [156].

Therefore, no particular heterojunction or Z-scheme can be seen as superior based on stated H_2_ evolution rates. However, composite materials containing CdS, BiVO_4_, or g-C_3_N_4_ often show high photocatalytic activity when combined with a suitable second semiconductor. Furthermore, new strategies like 2D/2D Z-schemes, the use of upconversion photoluminescent materials, or deliberate syntheses led to enhanced H_2_ production rates.

A benchmark was set in 2016 by Wang et al. who presented a photocatalyst sheet with a Z-scheme charge carrier transfer based on La- and Rh-codoped SrTiO_3_ and Mo-doped BiVO_4_ embedded into a gold layer [157]. A solar-to-H_2_ energy conversion of 1.1% and an apparent quantum yield of > 30% for pure water splitting was reported. Unfortunately, only in one third of the reviewed publications were apparent quantum yields stated. Values for solar-to-H_2_ energy conversion are also only given for a few cases.

Since different sacrificial agents (MeOH, TEOA, lactic acid, NaS, Na_2_SO_3_, etc.) and different concentrations of sacrificial agents were used to enhance H_2_ production rates, this caused additional problems for good comparability. Here, the need for sacrificial agents is viable for mechanistic studies and should be avoided when the aim to maximise hydrogen production is discussed.

In 2015, Horst Kisch discussed that apparent quantum yields cannot be compared in a quantitative way. Clear standards are needed to make apparent quantum yields and evolution rates to some extent comparable. Kisch proposed that *optimal rates* should be measured in one unique photoreactor at a given lamp intensity [158]. Common standards, including a standard photoreactor, are needed in the field of photocatalysis and will be inevitable at some point. These standards will not be perfectly suitable for every photocatalyst, but standardised measurements could establish a better comparability.

In terms of materials, in the past 5 years a clear trend toward direct Z-schemes has been recognisable. Heterojunctions have been discussed extensively for decades and are still prominent in photocatalysis research, but nowadays more attention is paid to Z-schemes because of their stronger redox capabilities. Z-schemes are now in the third generation, called direct Z-schemes, and have lost several of the drawbacks of their predecessors (redox mediated Z-schemes and all-solid-state Z-schemes) such as backward reactions and light-shielding problems.

Various new heterojunctions and Z-schemes have been reported in the last years. Overall, a number of trends can be identified that also leave room for new approaches and ideas:

Nanoparticles, nanosheets, and other 0D, 1D, 2D, or 3D structures are commonly used in all kinds of photocatalysts and in (direct) Z-schemes. However, mesoporous Z-schemes are rarely reported. In some of these reports, the mesoporous structure is not even discussed or further investigated [159, 160]. High specific surface areas, enhanced diffusion of reactants, and improved charge carrier transfer (for example because of an “antenna mechanism” [39]) would make mesoporous Z-schemes promising photocatalysts. Obviously, one mesoporous semiconductor (PS I or PS II) would not be capable of being in intimate contact with a bulk material since the contact area would be significantly reduced because of the pores. Two-dimensional materials such as nanosheets might even block pores, which would take away the advantages of a mesoporous semiconductor. Therefore, careful material design of macroporous/mesoporous heterojunctions might be a way to partially maintain a porous structure and achieve sufficient contact between two semiconductors. Another approach would be core-shell Z-schemes with a porous or incomplete outer shell. This might be a promising strategy, especially for semiconductors in the core that are prone to corrosion or other effects.

The 2D/2D type Z-schemes are probably the most interesting trend in Z-scheme photocatalysis research. Nonetheless, their synthesis is challenging, and large-scale synthesis protocols are lacking so far. Furthermore, 2D structures are mostly reserved to layered semiconductors. Effective synthetic methods need to be developed to produce 2D photocatalysts from non-layered semiconductors, as has been done for SrTiO_3_ for example [161].

In general, intimate contact seems to be crucial for Z-scheme charge carrier transport. Typical mechanical/physical methods, such as ball-milling, grinding, or simple mixing of the materials, are frequently used. However, composites assembled by these methods have no intimate interface and both semiconductors are easily detached from each other. Strong interaction is favoured by in-situ growth strategies, hydrothermal and solvothermal treatment, solid-state synthesis, ion-exchange, and electrospinning [77].

Interestingly, chemical deposition of a semiconductor was used to deliberately construct Z-schemes (g-C_3_N_4_/CdS) while photodeposition led to the formation of a type II heterojunction [95]. It is worth mentioning that the type II heterojunction exhibited higher photocatalytic activity. Deliberate construction of Z-schemes is also possible using photooxidation and photoreduction [96]. These techniques might be applicable to other semiconductors such as CeO_2_ in case of photooxidation and a variety of metal sulphides for photoreduction.

The field of photocatalysis has experienced a revival of certain materials. Doped SrTiO_3_, for example, was intensively studied for many years and was used in the probably most prominent example for a Z-scheme (SrTiO_3_:La,Rh/Au/BiVO_4_:Mo) [157]. The question therefore arises as to the extent to which other semiconductors could be optimised for improved charge carrier transfer in Z-schemes and heterojunctions. Oxygen/sulphur vacancies and mid gap states have been used in several Z-schemes [58, 145, 162–164]. Intrinsic defects and doping strategies could be applied to other semiconductors to further improve charge carrier transport in direct Z-schemes.

Black phosphorous is another semiconductor that research has focused on recently. For example, a black/red phosphorus multiphase heterojunction has been prepared and tested for water splitting [154, 155]. Furthermore, supported black phosphorus nanosheets as H_2_-evolving photocatalyst achieved 5.4% energy conversion efficiency, demonstrating the potential of black phosphorus-based materials [165].

Apart from improving existing Z-schemes by doping and new combinations of known semiconductors, new material classes have been introduced. Metal organic frameworks (MOFs) and more recently covalent organic frameworks (COFs) have been used in Z-scheme photocatalysts [104–106, 111, 151]. Other continuing trends are localised surface plasmon resonances (LSPR) and upconversion photoluminescent (UCPL) materials to improve the light absorption of photocatalysts in the visible and near IR range.

In summary, heterojunction and (direct) Z-scheme photocatalysts with multiple semiconductor combinations are still highly investigated in photocatalysis research for hydrogen generation, with different materials strategies addressing varying issues including light absorption over broad wavelength range and charge carrier separation. New types of materials classes such as MOFs and COF and 2D materials are included in heterojunctions and Z-schemes more frequently. Both remain extremely promising material designs and present unique advantages over single material photocatalysts for hydrogen generation; the recent trends and developments have been presented in this work. Although promising approaches such as the application of LSPR and UCPL materials as well as new material combinations, new syntheses, sophisticated morphologies, and improved doping strategies will inevitably make photocatalytic systems more complex, the increase in complexity is and will be the result of knowledge-based improvements [6] developing new and better absorber combinations for hydrogen generation, which might allow, one day, the large-scale production of green H_2_ from photocatalytic water splitting.

## Figures and Tables

**Table 1:**
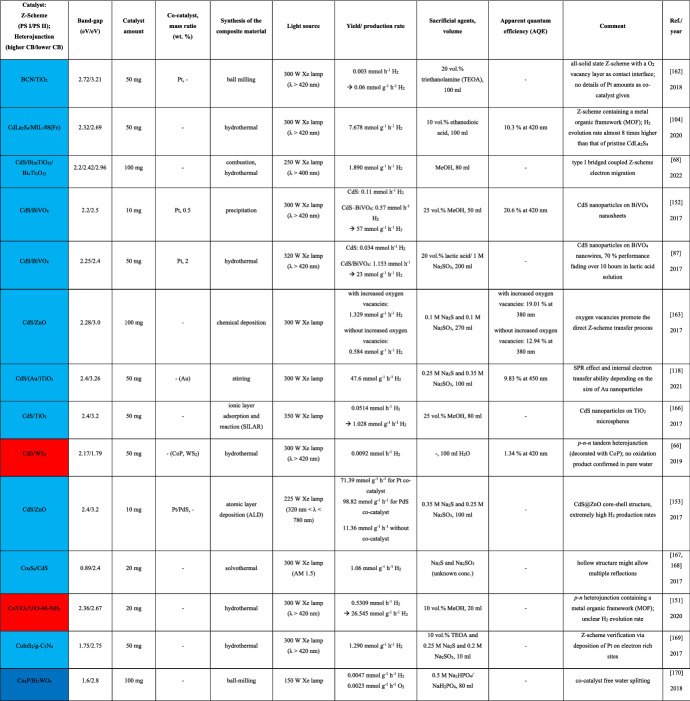

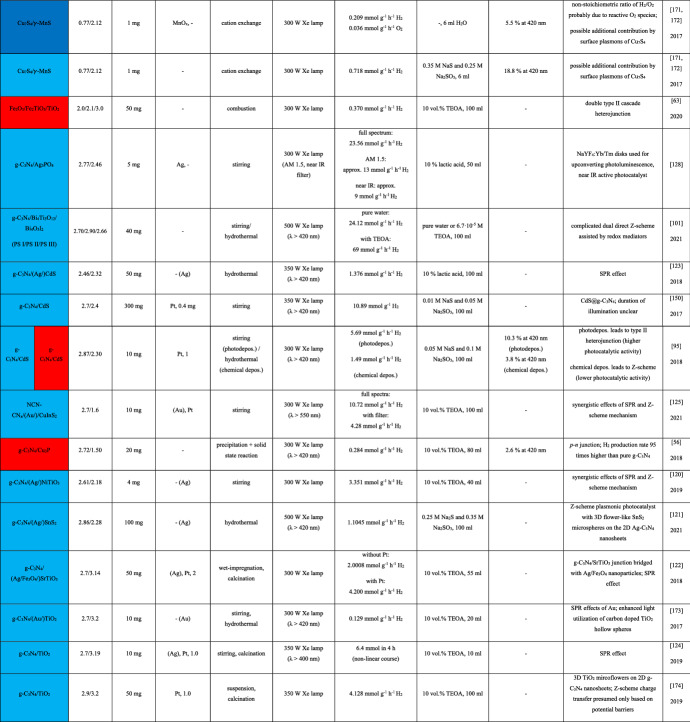

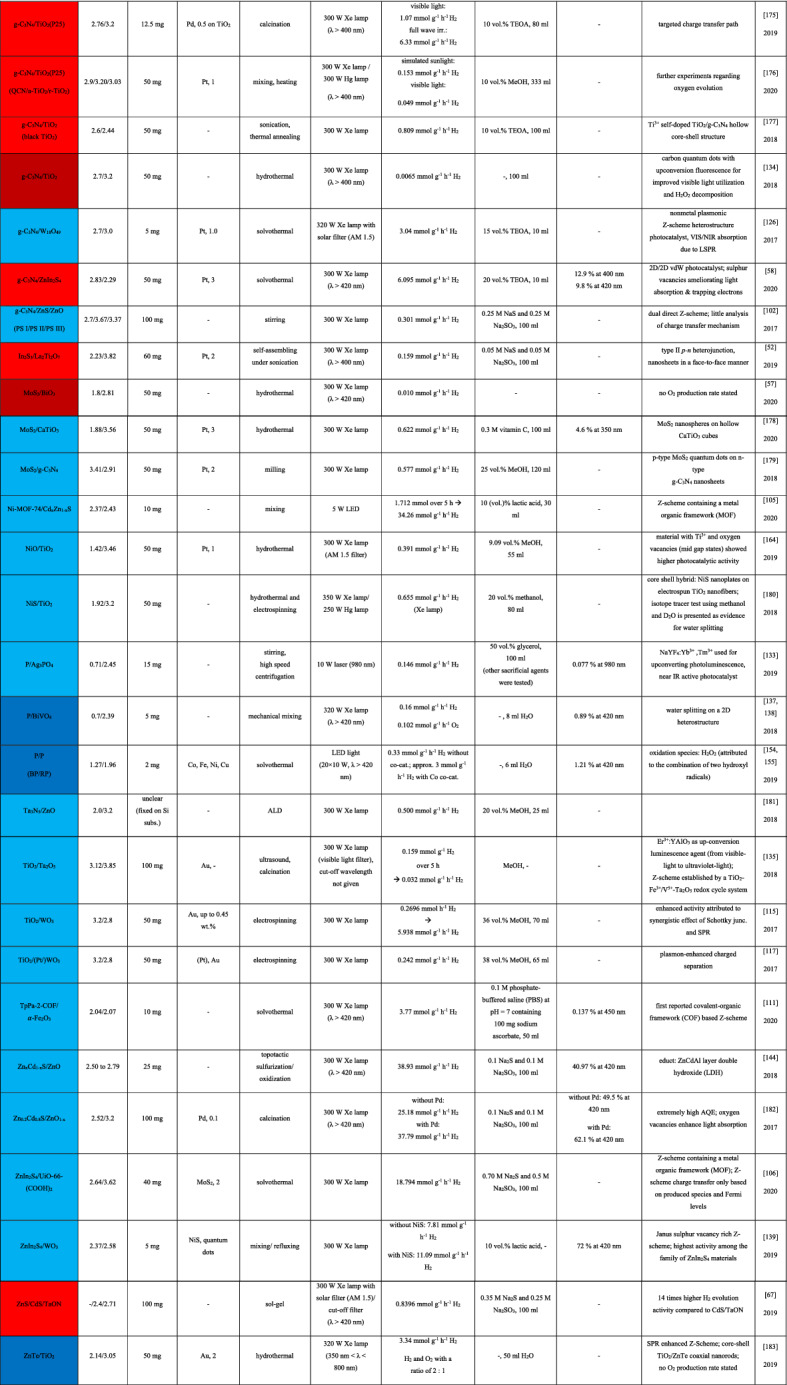
Recent heterojunction (red) and Z-scheme (blue) photocatalytic systems for H_2_ evolution (light colour) or water splitting (dark colour), respectively
